# Fungal Bergamotane Sesquiterpenoids—Potential Metabolites: Sources, Bioactivities, and Biosynthesis

**DOI:** 10.3390/md20120771

**Published:** 2022-12-08

**Authors:** Maan T. Khayat, Khadijah A. Mohammad, Abdelsattar M. Omar, Gamal A. Mohamed, Sabrin R. M. Ibrahim

**Affiliations:** 1 Department of Pharmaceutical Chemistry, Faculty of Pharmacy, King Abdulaziz University, Jeddah 21589, Saudi Arabia; 2 Center for Artificial Intelligence in Precision Medicines, King Abdulaziz University, Jeddah 21589, Saudi Arabia; 3 Department of Natural Products and Alternative Medicine, Faculty of Pharmacy, King Abdulaziz University, Jeddah 21589, Saudi Arabia; 4 Preparatory Year Program, Department of Chemistry, Batterjee Medical College, Jeddah 21442, Saudi Arabia; 5 Department of Pharmacognosy, Faculty of Pharmacy, Assiut University, Assiut 71526, Egypt

**Keywords:** bergamotanes, sesquiterpenoids, marine, fungi, biosynthesis, biological activities

## Abstract

The marine environment represents the largest ecosystem on the Earth’s surface. Marine-derived fungi are of remarkable importance as they are a promising pool of diverse classes of bioactive metabolites. Bergamotane sesquiterpenoids are an uncommon class of terpenoids. They possess diverse biological properties, such as plant growth regulation, phototoxic, antimicrobial, anti-HIV, cytotoxic, pancreatic lipase inhibition, antidiabetic, anti-inflammatory, and immunosuppressive traits. The current work compiles the reported bergamotane sesquiterpenoids from fungal sources in the period ranging from 1958 to June 2022. A total of 97 compounds from various fungal species were included. Among these metabolites, 38 compounds were derived from fungi isolated from different marine sources. Furthermore, the biological activities, structural characterization, and biosynthesis of the compounds are also discussed. The summary in this work provides a detailed overview of the reported knowledge of fungal bergamotane sesquiterpenoids. Moreover, this in-depth and complete review could provide new insights for developing and discovering new valuable pharmaceutical agents from these natural metabolites.

## 1. Introduction

Nature has substantially participated in the discovery of drugs for human remedial treatments since the beginning of mankind [1]. The marine environment, with more than 70% of the surface of the Earth, represents the largest ecosystem and is characterized by quite variable physicochemical parameters (e.g., limited light access, low temperature, high pressure, and high salinity) [2]. Among the various marine microbes, fungi are a superabundant and ecologically substantial component of marine microbiota [3]. Fungi are one of nature’s treasures that inhabit various environments on the earth’s surface, including the marine environment [4,5,6,7]. They play a growing relevant role in drug development and biomedicine research, either directly as drugs or indirectly as lead structures for bio-inspired drug synthesis [8,9,10,11,12]. In the last decades, natural product chemists and pharmacologists have turned their research interests to marine-derived fungi, which are renowned as a vast unexploited reservoir of metabolic diverseness and found to have the capability to produce structurally unique bio-metabolites [6,7,12,13,14,15,16]. Furthermore, research on fungi-derived metabolites has tremendously increased because of the need for compounds with potential economical values and pharmaceutical applications. Sesquiterpenes belonging to various classes, including hirsutane, alliacane, tremulane, bergamotane, drimane, etc., are reported from fungi [17,18,19]. The biosynthesis of their C15 skeleton from FPP (farnesyl pyrophosphate) was catalyzed by sesquiterpene synthases [19,20].

Among these metabolites, the bergamotane family represents an uncommon class of natural sesquiterpenes that includes bi-, tri-, or tetracyclic derivatives [19]. Bergamotane sesquiterpenoids having a bridged 6/4 bicyclic skeleton involved in an isopentyl unit are biosynthesized by fungi and plants [21,22]. Interestingly, polyoxygenated derivatives featuring a 6/4/5/5 tetracyclic framework represent a rare class of natural metabolites, and all polycyclic bergamotanes are mainly encountered in fungi [23,24,25,26]. Bergamotane sesquiterpenoids have been reported from various marine sources such as sponges, sea mud, deep-sea hydrothermal sulfide deposits, and sea sediments. These metabolites could gain the interest of chemists and biologists because of their unusual structural features and diversified activities, such as phytotoxicity, plant growth regulation, antimicrobial, anti-HIV, cytotoxic, pancreatic lipase inhibition, immunosuppressive, antidiabetic, and anti-inflammatory properties. It is noteworthy that no available work has addressed this class of sesquiterpenes in term of their sources, bioactivities, and biosynthesis. In the current work, the reported fungal bergamotane sesquiterpenoids ranging from 1958 to June 2022 have been listed. They have been classified according to their ring system, i.e., into bi-, tri-, or tetracyclic derivatives (Table 1). Additionally, their fungal sources, structural characterization, biosynthesis, and biological relevance have been provided. Moreover, some of their reported structural characteristics and methods of separation and characterization, as well as their structure–activity relation, are discussed.

Surveying their bioactivities may open a new research area for the synthesis of new agents from these metabolites by synthetic and medicinal chemists. The literature search for the reported data was performed using diverse databases and publishers, including Web of Science, Google Scholar, PubMed, Scopus, SciFinder, Wiley, SpringerLink, and ACS Publications, using specific keywords (bergamotane, marine, fungi, biosynthesis, and biological activities).

## 2. Structural Assignment and Stereochemistry Determination

A total of 97 metabolites have been separated from various fungal source extracts using different chromatographic techniques and characterized by NMR, MS, and IR spectral analyses as well as chemical derivatization. The relative configuration of these metabolites was established using NOESY or ROESY spectral analyses. Various studies reported the assigning of their absolute stereochemistry using total synthesis [53,54], Mosher’s method [26], X-ray diffraction, chemical conversion [34,43,55], and ECD analyses [31]. The reported metabolites have been categorized into bi-, tri-, and tetracyclic derivatives.

## 3. Biological Activities of Bergamotane Sesquiterpenoids

Various reported studies revealed the assessment of bergamotane sesquiterpenoids for diverse bioactivities, including plant growth regulation, phototoxic, antimicrobial, anti-HIV, cytotoxic, pancreatic lipase inhibition, antidiabetic, anti-inflammatory, and immunosuppressive, which were summarized in this work (Table 2). Additionally, the reported structure–activity relation was included.

### 3.1. Anti-Inflammatory Activity

NO (nitric oxide) is a substantial pro-inflammatory mediator, and its excessive production is accompanied with various inflammatory illnesses; therefore, it possesses a remarkable role for regulating immune responses and inflammation [56]. NO production inhibitors may represent the potential capacity for treating various inflammatory disorders. Thus, further research for fungal metabolites must be conducted to discover novel anti-inflammation agents.

The epigenetic chemical manipulation of *Eutypella* sp. derived from deep-sea hydrothermal sulfide deposit by co-treatment with SBHA (histonedeacetylase inhibitor, suberohydroxamic acid) and 5-Aza (DNA methyltransferase inhibitor, 5-azacytidine) was shown to activate a biosynthetic sesquiterpene-linked gene cluster [35]. From elicitor-treated cultures EtOAc extract, eutypeterpenes A–F (**18**–**22** and **28**) along with xylariterpenoids A (**16**) and B (**17**) were purified using SiO_2_/RP-18/HPLC that were identified by spectral analyses, as well as by using chemical conversion, X-ray diffraction, ECD, and calculated NMR for configuration assignments.

**Figure 1:**
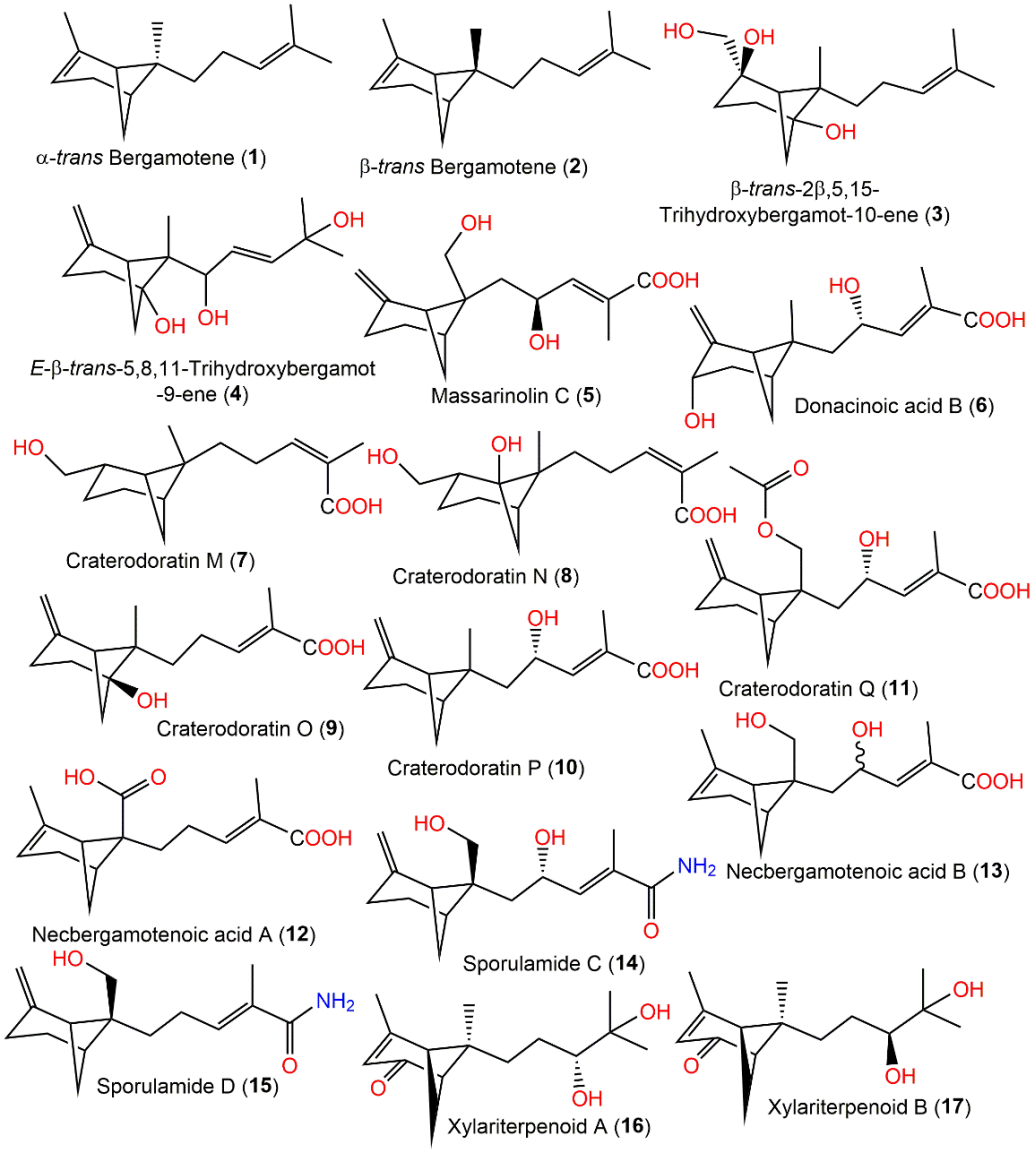
Structures of bicyclic bergamotane sesquiterpenoids (**1**–**17**).

Eutypeterpene A (**28**) is the first bergamotene sesquiterpene incorporating a dioxolanone moiety. These metabolites were assessed for their NO production inhibitory capacity induced by LPS-(lipopolysaccharide) in RAW 264.7 macrophages [35]. The results indicated thatcompound **18** and **19** (IC_50_ 13.4 and 16.8 μM, respectively) displayed more effectiveness than quercetin (IC_50_ of 17.0 μM), whereas other metabolites had noticeable potentials (IC_50_ values ranged from 18.7 to 24.3 μM) with weak cytotoxic capacities (IC_50_ > 100 μM). A structure–activity study revealed that the analog with a triol unit (**18**) at the side chain was more effective than compound **16**, **17**, and **19** with a diol unit, which were more potent than compound **20**, **21**, and **28** with one hydroxy group. Furthermore, the α,β-unsaturated ketone unit (as in compound **21** and **22**) and the OH-linked carbon configuration also affected the activities (**16** versus **17**) [35] (Figure 1).

**Scheme 1:**
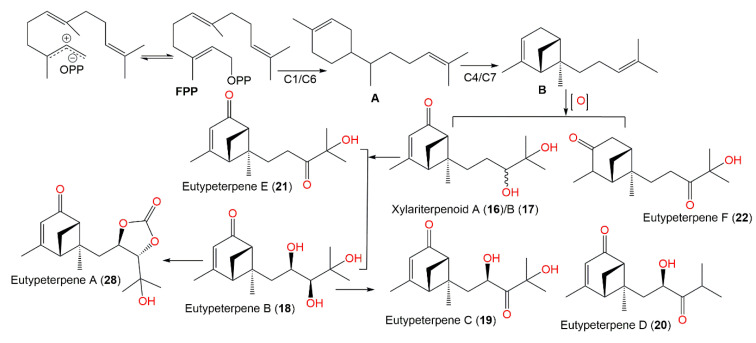
Biosynthetic pathway of eutypeterpenes A–F (compounds **18**–**22** and **28**) [35].

Biogenetically, compounds **18**–**22** are derived from FPP that performs a 1,6-cyclization to produce bisabolane (**A**). The 4,7-cyclization of **A** generates bergamotane (**B**), which further generates **18**–**22** via diverse oxidation and reduction processes. Additionally, compound **28** is formed from **18** by carbonate incorporation [35] (Scheme 1).

**Figure 2:**
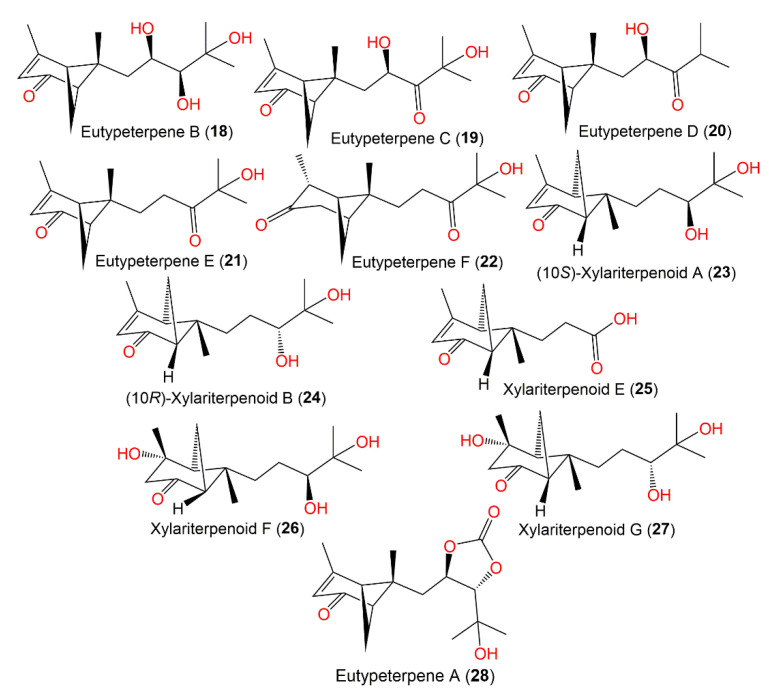
Structures of bicyclic bergamotane sesquiterpenoids (compounds **18**–**28**).

The deep-sea-isolated *Graphostroma* sp. MCCC3A00421 associated with the Atlantic Ocean hydrothermal sulfide deposits biosynthesized new bergamotane sesquiterpenoids: (10S)-xylariterpenoid A (**23**), (10R)-xylariterpenoid B (**24**), xylariterpenoid E (**25**), xylariterpenoid F (**26**), and xylariterpenoid G (**27**), which were purified using SiO_2_/OSD/Sephadex LH-20/RP-18 CC and preparative TLC. They were characterized by extensive spectral data, and their absolute configuration was established by ECD, Cu-Ka-single-crystal X-ray diffraction, and modified Mosher’s method analyses. Compound **25** is trinor-bergamotane. Compounds **23**, **26**, and **27** revealed moderate inhibition potentials (IC_50_s of 86, 85, and 85 μM, respectively) of NO production in LPS-stimulated RAW264.7 macrophages compared with aminoguanidine (IC_50_ of 23 μM). It was noted that bergamotane moiety’s 10S configuration obviously boosted the activity as in compound **23** (10S, IC_50_ of 85 μM) versus compound **24** (10R, of IC_50_ 230 μM) (Figure 2) [34].

### 3.2. Phytotoxic Activity

**Figure 3:**
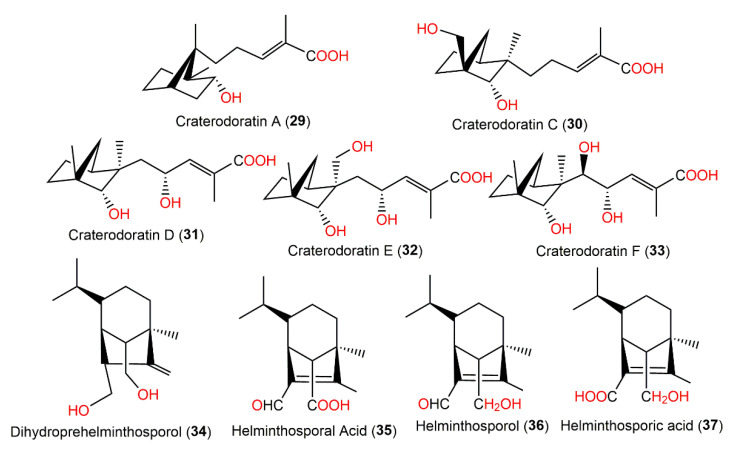
Structures of the bicyclic bergamotane sesquiterpenoids (compounds **29**–**37**)**.**

Prehelminthosporol (**38**) and dihydroprehelminthosporol (**34**) along with compounds **35**–**37**, **39**, **40**, and **43** were separated by SiO_2_, flash CC, and preparative TLC from the EtOAc extract of the *Bipolaris* species, which is a *Sorghum halepnse* (Johnson grass) pathogen (Figure 3). These metabolites were assessed for their phytotoxic potential towards *Sorghum bicolor* (Sorghum) and *Sorghum balepense* (Johnson grass) in leaf spot assays [36,37]. Compounds **34**, **38**, and **39** produced similar lesions to those caused by the fungus in the field. The lesions appeared as a reddish-brown area (0.3–0.5 cm diameter) surrounded by a black circle with an outer chlorotic zone. Compounds **34** and **38** (concentration of 25 μg/5 μL) had comparable toxic effectiveness, while compound **38** maintained its effect at a lower concentration of 2.5 μg/5 μL; meanwhile, the other compounds were non-toxic [36,37]. Victoxinine was also toxic to cereals in the order of oats > rye and barley > wheat > sorghum in a root inhibition assay [37]. The phytotoxic influence of compounds **34** and **38**–**40** versus sorghum, corn, bent-grass, sickle-pod, and morning glory was also assessed in leaf spot assays. Moreover, victoxinine caused a water-soaked translucent appearance with defined irregular necrotic edges. It is worth mentioning that 3-deoxyanthocyanidins are sorghum stress response metabolites (phytoalexins), which were accountable for the red wound response. Compounds **34**, **38**, and **39** were elicitors of a very strong reddening compared with the wounding-produced reddening, but compound **40** did not elicit a sorghum phytoalexin response. In bent grass and corn, compounds **34** and **38**–**40** produced a light-brown area limited by a chlorotic region, whereas in sickle pod and morning glory, they showed necrotic lesions that extended at high concentrations beyond the under-drop area. It is noteworthy that compound **38** was the most toxic compound versus all tested plants except for the morning glory [37].

**Figure 4:**
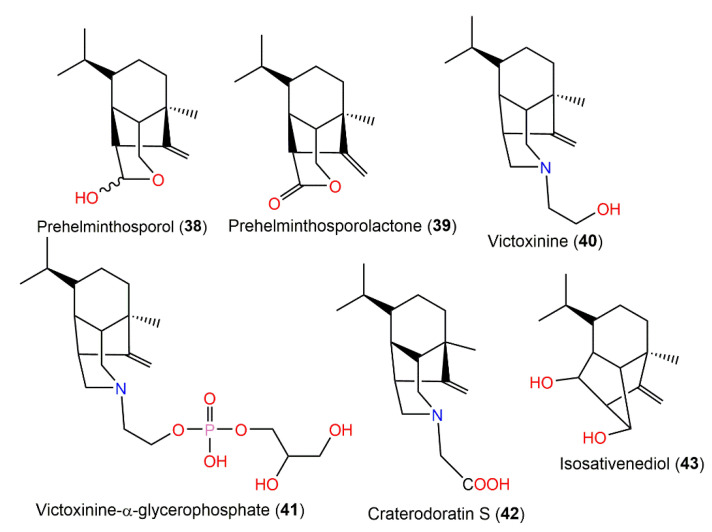
Structures of tricyclic bergamotane sesquiterpenoids (compounds **38**–**43**)**.**

Helminthosporium victoriae, the causative agent of oats Victoria blight disease yielded phytotoxins, victoxinine (**40**) and victoxinine α-glycerophosphate (**41**), which were separated from its diethyl ether extract using Sephadex LH-20 and SiO_2_ CC and detected on the TLC plate by giving a blue color with 5% vanillin:H_2_SO_4_ [41] (Figure 4). The existence of α-glycerophosphate moiety was established by coupling between the phosphorous and carbon. Compound **40** completely prohibited the root growth of toxin-susceptible and toxin-resistant oats (concentration of 2.5 × 10^−4^ M); it was ≈ 7500 times more toxic for susceptible plants on a weight basis, while its toxicity for resistant plants was nearly similar, suggesting a role of the victoxinine moiety on the toxicity [38,39,41]. On the other side, compound **41** (concentration of 100 µg/mL) demonstrated little or no growth inhibition effectiveness on either susceptible or resistant oats [41]. 

### 3.3. Anti-HIV Activity

**Figure 5:**
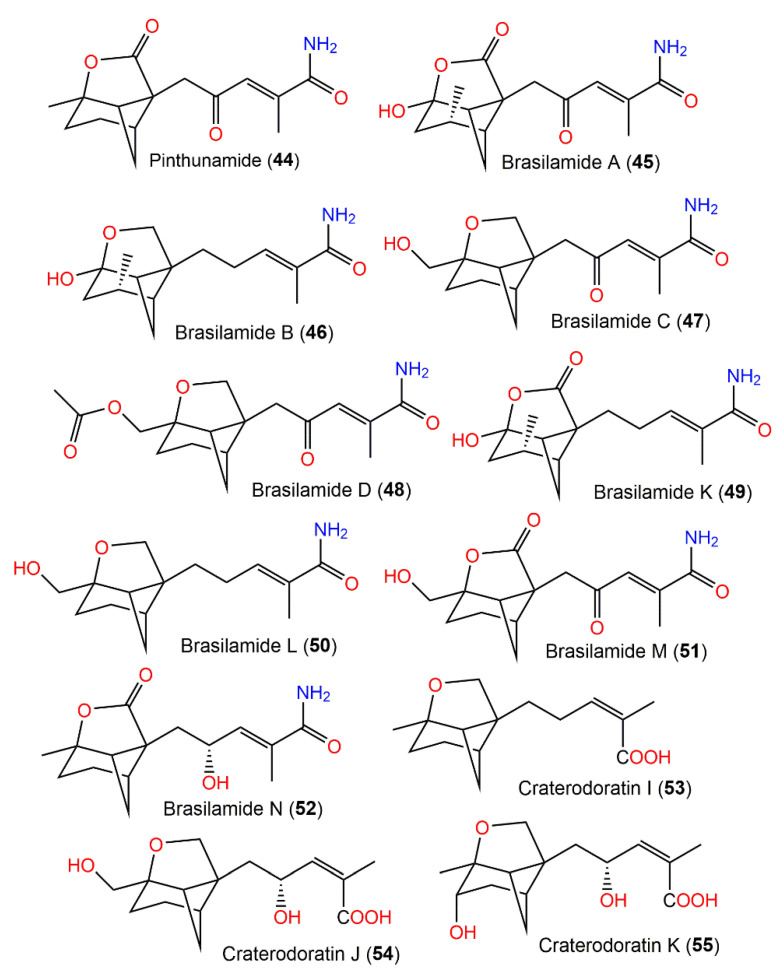
Structures of tricyclic bergamotane sesquiterpenoids (**44**–**55**)**.**

From *Paraconiothyrium brasiliense*, new tricyclic sesquiterpenoids, brasilamides A–D (**45**–**48**) and the formerly reported pinthunamide (**44**), were separated from the culture’s EtOAc extract utilizing SiO_2_/Sephadex LH-20 CC and HPLC. Their structures were established using NMR and X-ray analyses (Figure 5). Compounds **45** and **46** are rare metabolites having a 4-oxatricyclo[3.3.1.0^2,7^]nonane moiety with a tetrahydro-2*H*-pyrone or a tetrahydro-2*H*-pyran linked with bicyclo[3.1.1]heptane ring at C-5 and C-2, whereas compounds **47** and **48** are analogs of **44**, possessing an unprecedented 9-oxatricyclo[4.3.0.0^4,7^]-nonane core.

**Scheme 2:**
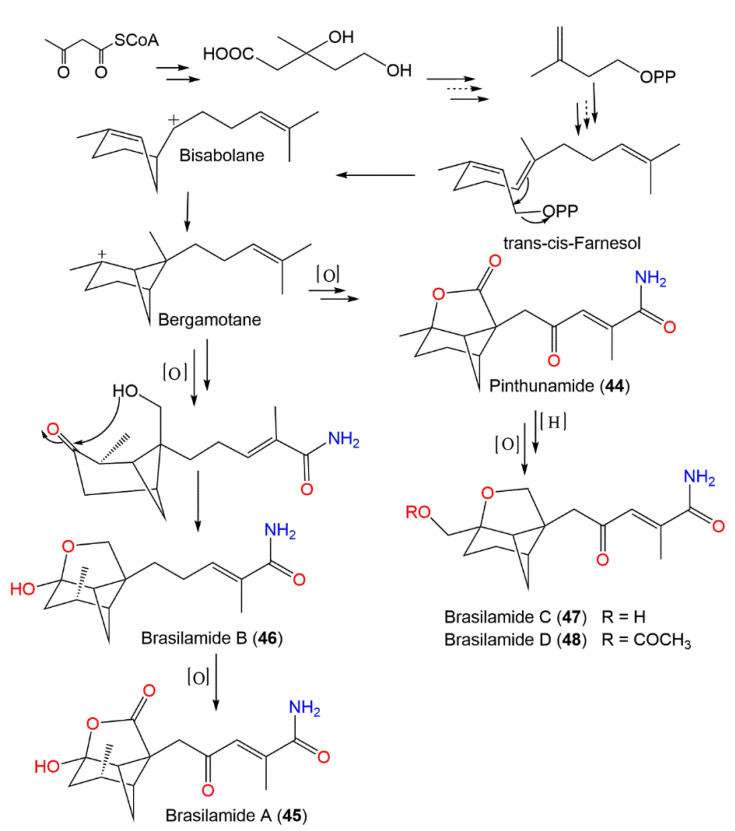
Biosynthetic pathways of brasilamides A–D (**45**–**48**) [43].

The differences of the above-mentioned compounds from **44** were the existence of a tetrahydrofuran moiety connected to the bicycle[3.1.1]heptane unit instead of γ-lactone ring, as well as different C-10 substituents. Compounds **45**–**48** demonstrated inhibitory effectiveness (EC_50_s of 108.8, 57.4, and 48.3 µM, respectively) versus HIV-1 replication in C8166 cells compared with indinavir sulfate (EC_50_ of 8.2 nM) [43]. Biogenetically, they were derived from the mevalonate/*trans*-*cis*-farnesol/bisabolene/bergamotane pathway (Scheme 2).

### 3.4. Immunosuppressive Activity

Immunosuppressants are drugs that prohibit body immunity and are principally utilized in organ transplantation to overcome rejection and in auto-immune illnesses [57]. Currently, many immunosuppressive agents act by prohibiting T-cell proliferation; however, there is no new, safe, and efficient immune-suppressive agent that prohibits B-cell proliferation [58].

**Figure 6:**
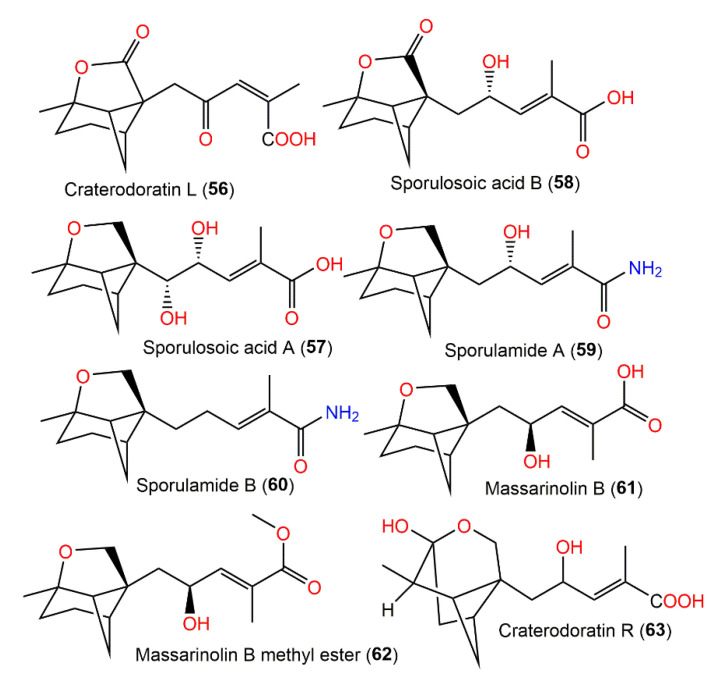
Structures of tricyclic bergamotane sesquiterpenoids (**56**–**63**).

Dai et al. separated eighteen bergamotane sesquiterpenoids from the EtOAc extract of *Craterellus ordoratus*: craterodoratins A–R (**7**–**11**, **29**–**33**, **53**, **55**, **56**, **63**–**65**, and **71**) and a new victoxinine derivative, craterodoratin S (**42**), along with the previously isolated **5**, **61**, **77**, and **88** by SiO_2_/RP-18/Sephadex LH-20/preparative HPLC (Figure 6) [30].

**Figure 7:**
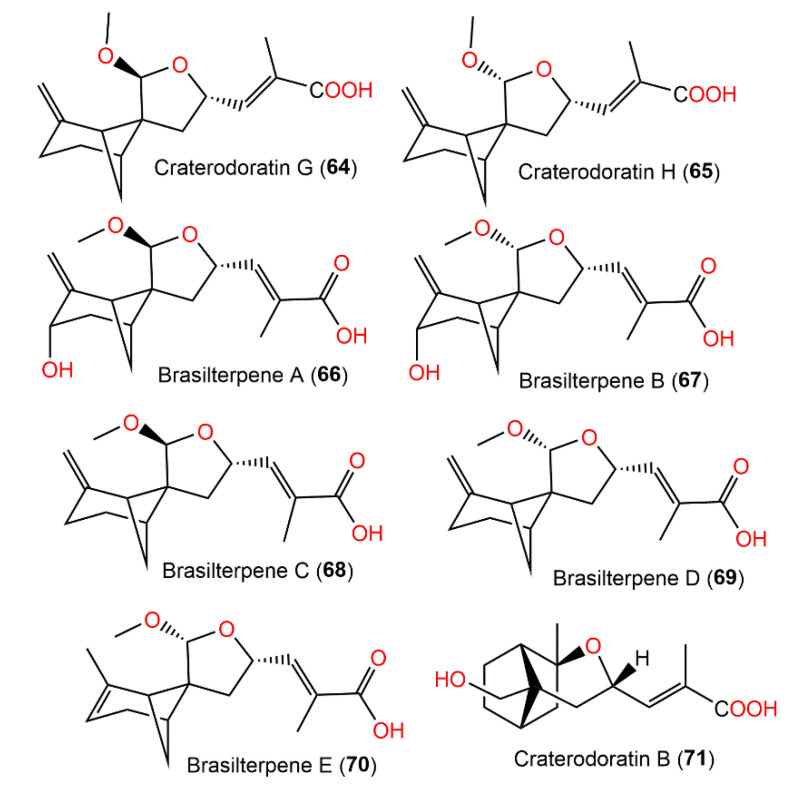
Structures of tricyclic bergamotane sesquiterpenoids (**64**–**71**)**.**

Their structures with absolute configurations were established by spectral, X-ray diffraction, and ECD analyses and NMR calculations. Compounds **29** and **71** possess a rare skeleton, where the C-14methyl in **71** showed a further 1,2-migration. On the other hand, compounds **7**–**11**, **53**, **55**, **56,** and **63**–**65** belong to β-pinene derivatives that produced **30**–**33** through an alkyl migration (Figure 7). Compounds **7**–**10**, **30**, **42**, **55**, **61**, and **88** demonstrated potent inhibitory potential versus LPS-caused B lymphocyte cell proliferation (IC_50_s ranged from 0.67 to 22.68 μM) in BALB/c mice compared with cyclosporin A (IC_50_ of 0.47 μM), where compound **61** (IC_50_ 0.67 μM) had the most potent effectiveness. Moreover, compounds **11** and **61** possessed inhibition (IC_50_s of 31.50 and 0.98 μM, respectively) on T lymphocyte cells proliferation induced by ConA (concanavalin A) compared with cyclosporin A (IC_50_ 0.04 μM). Structurally, it was noted that the *α*,*β*-unsaturated-carboxylic acid unit could be the key functional group for the immunosuppressive potential of these metabolites. Furthermore, compounds **61** and **7**–**10** with a *β*-pinene main core had a wider range of bioactivities [30].

### 3.5. Antimicrobial Activity

**Figure 8:**
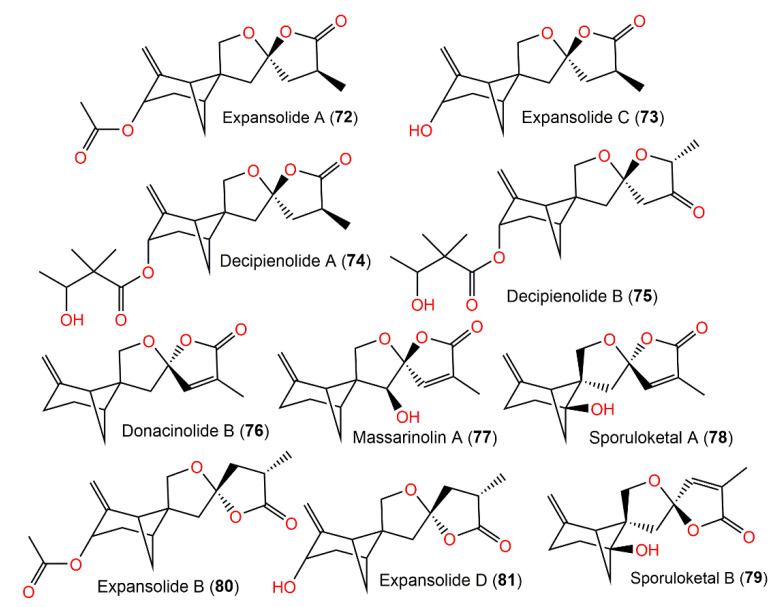
Structures of tetracyclic bergamotane sesquiterpenoids (**72**–**79**).

From *Podospora decipiens*, two new tetracyclic sesquiterpenoids, decipienolides A (**74**) and B (**75**), were separated from the EtOAc extract by SiO_2_ CC and HPLC analyses. They were obtained as a mixture of inseparable epimers, having a 3-hydroxy-2,2-dimethylbutyric acid sidechain as elucidated by an NMR analysis (Figure 8). The **74**/**75** mixture had an antibacterial influence versus *B. subtilis* (inhibition zone diameter of 9–10 mm, concentration of 200 µg/disk). Neither of them demonstrated capacity versus *Ascobolus furfuraceus* NRRL6460, *Sordaria fimicola* NRRL6459, and *C. albicans* ATCC90029 [24]. Donacinolides A (**82**) and B (**76**) (concentration of 50 μg/mL) revealed weak inhibition versus *Salmonella enterica* subsp. *enterica* (inhibition rates of 24.3, 23.9, and 26.2%) in the microdilution assay [32]. Furthermore, there were no observed antibacterial activity for purpurolides B (**83**) and C (**84**) (concentration of 50 μM) versus *E. coli* ATCC25922, *M. smegmatis* mc2155 ATCC70084, *S. aureus* ATCC25923, and *S. epidermidis* ATCC12228 [47].

### 3.6. Pancreatic Lipase Inhibition

**Figure 9:**
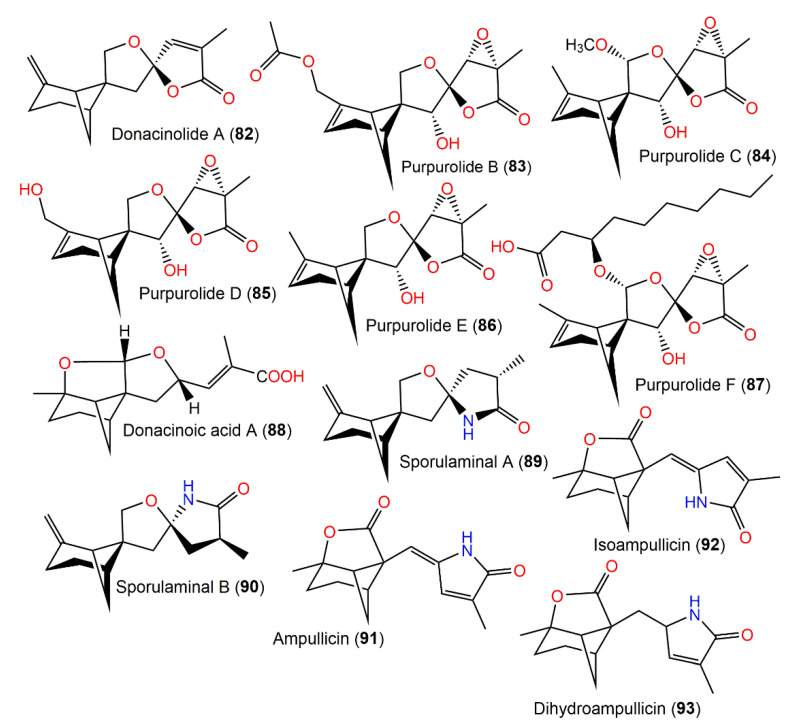
Structures of tetracyclic bergamotane sesquiterpenoids (**80**–**93**).

Purpurolides B (**83**) and C (**84**) are new 6/4/5/5-tetracyclic sesquiterpenoids that were separated from *Penicillium purpurogenum* IMM003 cultures by SiO_2_/RP-18/preparative HPLC analysis. The structures and configurations of compounds **83** and **84** were established using spectral and X-ray analyses as well as ECD and GIAO NMR data calculations (Figure 9).

**Scheme 3:**
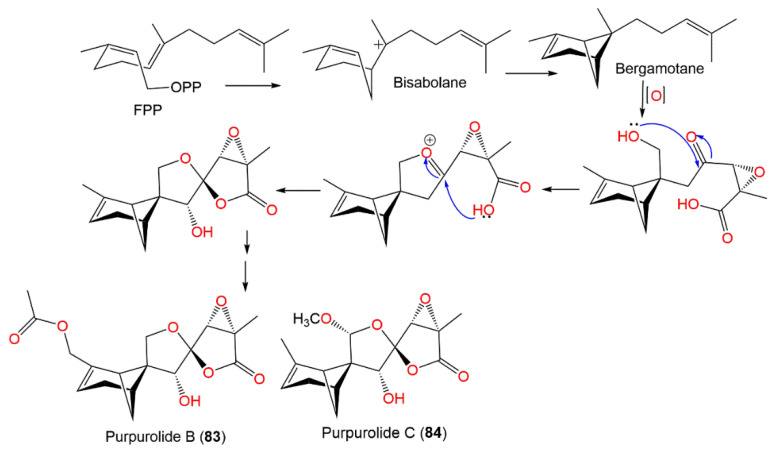
Biosynthetic pathway of purpurolides B and C (**83** and **84**) [47].

Compounds **83** and **84** demonstrated potent pancreatic lipase inhibition (IC_50_s of 5.45 and 6.63 μM, respectively), compared with kaempferol (IC_50_ of 1.50 μM) [47]. These compounds were possibly biosynthesized via numerous the cyclization and enzyme-catalyzed oxidation of FPP (farnesyl pyrophosphate), leading to four- and six-membered rings and the formation of two five-membered heterocyclic rings (Scheme 3) [47].

Xia et al. separated from *Penicillium purpurogenum* IMM003 purpurolides D–F (**85**–**87**), which are new polyoxygenated 6/4/5/5-tetracyclic bergamotanes, using SiO_2_/Sephadex-LH-20/RP-18 CC and preparative HPLC processing [48]. Their elucidation was accomplished using spectral ^13^C NMR calculations coupled with DP^4+^ probability and ECD analyses. Compound **87** had potent pancreatic lipase inhibition potential (IC_50_ of 1.22 μM) compared with kaempferol (IC_50_ of 1.50 μM) and orlistat (IC_50_ of 0.75 μM), whereas compounds **85** and **86** (IC_50_s of 6.50 and 7.88 μM, respectively) were five or six-fold less powerful than **87**, revealing that the C-14 hydroxylated decanoic acid moiety increased the potency [48]. Therefore, polyoxygenated bergamotanes could be viable candidates as pancreatic lipase inhibitors for further clinical development [48].

### 3.7. Antidiabetic Activity

**Scheme 4:**
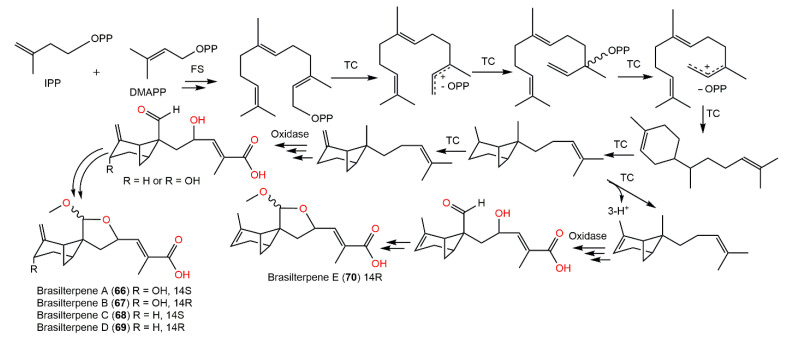
Biosynthetic pathway of brasilterpenes A-E (**66**–**70**) [45]. IPP: isopentenyl diphosphate; FS: farnesyl synthase; NPP: nerolidyl diphosphate; TC: terpenyl cyclase; DMAPP: dimethylallyl diphosphate; FPP: farnesyl diphosphate.

From the deep sea-derived *Paraconiothyrium brasiliense* HDN15-135 EtOAc extract, new bergamotane sesquiterpenoids, brasilterpenes A-E (**66**–**70**), featuring an uncommon 6/4/5-tricyclic ring system, were separated by SiO_2_/RP-18/Sephadex LH-20/HPLC and assigned by diverse NMR analyses and X-ray diffraction, ECD, and DFT-NMR (density functional theory calculations of nuclear magnetic resonance) data [45]. Their hypoglycemic potential was estimated utilizing β-cell-ablated zebrafish larvae. Compounds **66** and **68** (concentration of 10 μM) remarkably lessened the glucose level down to 449.3 and 420.4 pmol/larva respectively, compared with the β-cell-ablated group (Teton+) (glucose level of 502.8 pmol/larva) and rosiglitazone (glucose level 395.6 pmol/larva) with no toxic influence on zebrafish larvae up to 200 μM. It was found that compounds **66** and **68** notably minimized free blood glucose in vivo in hyperglycemic zebrafish by suppressing gluconeogenesis and improving insulin sensitivity, which revealed that compound **68** had promising antidiabetic potential [45]. The structure–activity study revealed that the activity may be linked to the C-14 *S-*configuration of compounds **66** and **68**, which represent the main structural difference from **67** and **69.** The existence of C-3-OH may weaken the influence in **68** versus **66**; however, the △^2^ endocyclic double bond may enhance the potential in **70** versus **69** [45]. Therefore, compound **68** may provide a scaffold for hypoglycemic drug development. Compounds **66**–**70** are also biosynthesized by the FPP pathway (Scheme 4). The cyclization of FPP via NPP (nerolidyl diphosphate) followed by a bisabol intermediate yields the bergamotane skeleton. These compounds are created by further oxidation, 9-OH-nucleophilic attack, and methylation processes. Because of the nucleophilic attack direction flexibility during the furan ring formation, compounds **66**–**69** appeared as C14-epimers in pairs [45] (Scheme 4).

Ying et al. isolated two new derivatives, expansolides C (**73**) and D (**81**), in addition to **72** and **80** from the plant pathogen *Penicillium expansum* ACCC37275 [46]. In an α-glucosidase inhibition assay; the **73**/**81** epimeric mixture (ratio 2:1) possessed a more powerful effectiveness (IC_50_ of 0.50 mM) compared with acarbose (IC_50_ 1.90 mM), while the **72**/**80** epimeric mixture possessed no apparent potential. It was assumed that the acetyl group in compounds **72** and **80** impeded their binding with the α-glucosidase, resulting in loss of activity [46].

### 3.8. Plant Growth Regulation

**Scheme 5:**
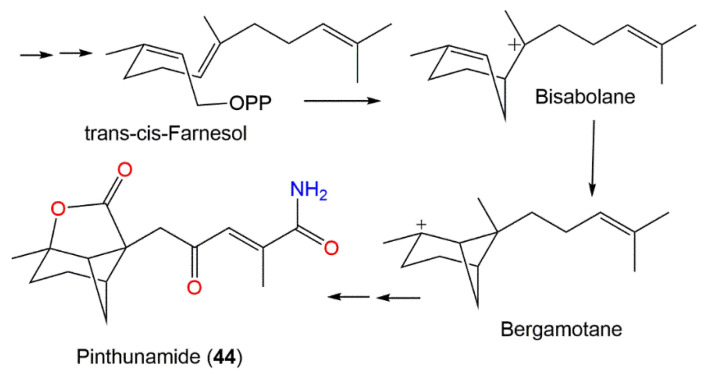
Biosynthesis pathway of pinthunamide (**44**) [42].

Kimura et al. purified the tricyclic amide sesquiterpenoid pinthunamide (**44**) from the acetone extract of *Ampulliferina* sp. at pH 2.0 utilizing SiO_2_ and sephadex LH20 CC processing as well as crystallization from EtOAc extract, which gave positive NH_2_OH-HCI-FeCl_3_ and KMnO_4_ reactions [42]. The compound was assigned by X-ray diffraction and NMR methods. Its plant growth regulation effectiveness was evaluated using a lettuce seedling assay, where it (dose 300 mg/L) produced a 150% root growth acceleration over the control seedlings (100%) while scarcely influencing the hypocotyl elongation at the tested concentrations [42]. Its structure combined a unique configuration of six-, five-, and four-membered rings that was proposed to be biosynthesized via the mevalonate/*trans*-*cis*-farnesol/bisabolene/bergamotane pathway (Scheme 5) [42].

**Figure 10:**
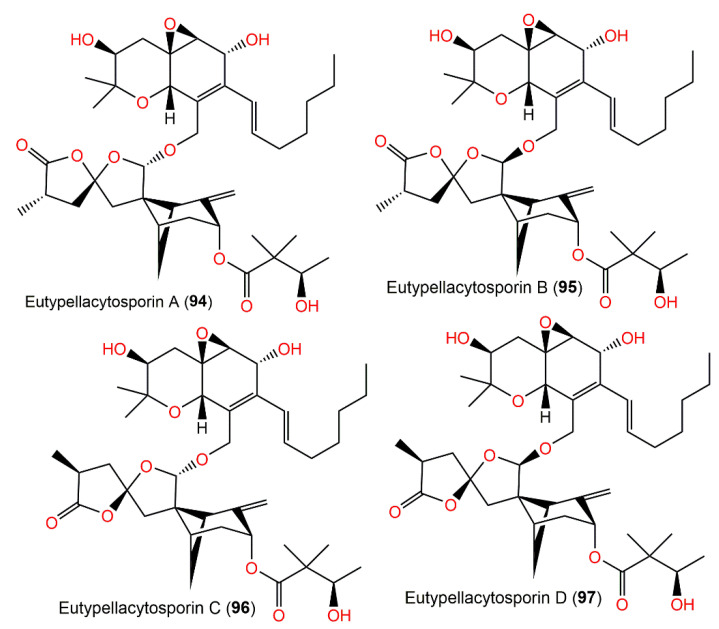
Structures of tetracyclic bergamotane sesquiterpenoids (**94**–**97**).

Furthermore, in 1990, Kimura et al. purified another two new plant growth regulators, ampullicin (**91**) and isoampullicin (**92**) from *Ampulliferina* sp. No. 27 associated with *Pinus thunbergii* dead tree by SiO_2_ CC utilizing benzene:acetone as an eluent [50] (Figure 10). They were stereoisomers that had γ-lactam rings. Additionally, they (doses of 300 and 30 mg/L) were shown to promote lettuce seedling root growth by 200% over the control lettuce seedlings (100%) [50]. In 1993, the same group separated a minor metabolite, dihydroampullicin (**93**), characterized by the absence of the C8-C9 double bond. The compound promoted a 160% growth rate in lettuce seedling roots (dose of 300 mg/L) compared with the control; however, it had no influence on the hypocotyl growth, indicating that the C8-C9-double bond (C8-C9) was substantial in lettuce seedlings` root growth [51]. Bermejo et al. reported the synthesis of (**+**)**−91** and **92** from (*R*)-(-)-carvone with a 4.5% overall yield using a stereo-selective 18-step sequence application [59]. The EtOAc extract of *Aspergillus fumigatus* Fresenius separated from leaf litter yielded expansolides A (**72**) and B (**80**). They had 2*S*/4*S*/6*S*/7*R*/9*R*/11*S* and 2*S*/4*R*/6*S*/7*R*/9*R*/11*S*, respectively, based on modified Mosher’s method. The compounds noticeably prohibited etiolated wheat coleoptiles growth by 100% and 59% at 10^−3^ M and 10^−4^ M solution compared with LOGRAN (commercial herbicide) (%inhibition of 80 and 42%) at the same concentrations [26].

### 3.9. Cytotoxic Activity

Compounds **3** and **4**, which were new β-bergamotane sesquiterpenoids, were separated by SiO_2_/RP-18/HPLC from the marine-associated *Aspergillus fumigatus-*YK-7 EtOAc extract. Their antiproliferative effects on the U937 and PC-3 cell lines were measured in vitro in an MTT assay. Compound **4** revealed a weak growth inhibition capacity (IC_50_ of 84.9 µM) versus the U937 cell line, while **3** had no activity (IC_50_ > 100 µM) compared with doxorubicin hydrochloride (IC_50_ of 0.021 µM). On the other sides, both had no effect versus PC-3 cells [29]. Wu et al. reported the separation of two new derivatives, xylariterpenoids A and B (**16** and **17**), from the EtOAc extract of *Xylariaceae* fungus by Sephadex LH-20/ODS CC and reversed-phase HPLC processing [33]. Their structures and stereo-configuration were proved utilizing NMR and CD methods. They are C-10 epimers having 2S/6S/7S/10R and 2S/6S/7S/10S configurations, respectively. Unfortunately, they (IC_50_ > 40 μM) exhibited no cytotoxic potential versus HL-60, MCF-7, SMMC-7721, A-549, and SW480 in an MTT assay [33].

From *Paraconiothynium brasiliense* Verkley, new bergamotane sesquiterpenoids brasilamides K-N (**49**–**52**), featuring 4-oxatricyclo-(3.3.1.0^2,7^)-nonane (as in **49**) and 9-oxatricyclo-(4.3.0.0^4,7^)-nonane (as in **50**–**52**) skeletons in addition to the formerly reported brasilamides A and C (**45** and **46**), were purified from the fungus scale-up fermentation cultures using SiO_2_/Sephadex LH-20/HPLC processing. They were elucidated via NMR analyses and compound **52**’s configuration was assured using modified Mosher’s method. Compound **49** is a **45**-hydrogenated analog that has a tetrahydro-2H-pyrone unit linked at C-2 and C-5 to the bicyclo(3.1.1)heptane framework, forming a 4-oxatricyclo-(3.3.1.0 ^2,7^)-nonane skeleton, whereas compounds **50**–**52** displayed unusual 9-oxatricyclo-(4.3.0.0 ^4,7^)-nonane skeletons. Compounds **50** and **51** are hydrogenated and oxygenated derivatives of **46**, respectively, while **52** differed from **46** by having a C-8-carbonyl, C-1-methyl, and C-12 hydroxyl group instead of methylene, oxy-methylene, and ketone carbonyl, respectively. These metabolites (concentration of 50 µM) possessed no potential versus A549, A375, MCF-7, CNE1-LMP1, EC109, MGC, PANC-1, and Hep3B-2 in the MTS assay [44].

*Montagnula donacina* (edible mushroom) biosynthesized rare tetracyclic bergamotane sesquiterpenoids, donacinolides A (**82**) and B (**76**) and donacinoic acids A (**88**) and B (**6**), which were separated using SiO_2_ CC/Sephadex LH-20 CC/HPLC processing and were characterized using spectroscopic data, X-ray diffraction analysis, and computational methods. Compounds **76** and **82** are C9 epimers with a spiroketal moiety having 1S/5S/6S/9R and 1S/5S/6S/9S configurations, respectively, whereas **88** and **6** exhibited α,β-unsaturated carboxylic acid moiety and had 1R/2R/5S/6S/9S/14S and 1R/3S/5R/6R/9S configurations, respectively. These metabolites lacked a marked cytotoxic potential (IC_50_ > 40 μM) versus HL-60, SW480, A549, SMMC-7721, and MCF-7 [32].

In addition, purpurolides B (**83**) and C (**84**) had no cytotoxicity versus M14, HCT-116, U87, A2780, BGC-823, Bel-7402, and A549 [47], whereas compounds **85**–**87** (concentration of 50 μM) were inactive versus HCT-116, BGC-823, and Bel-7402 cell lines [48].

**Scheme 6:**
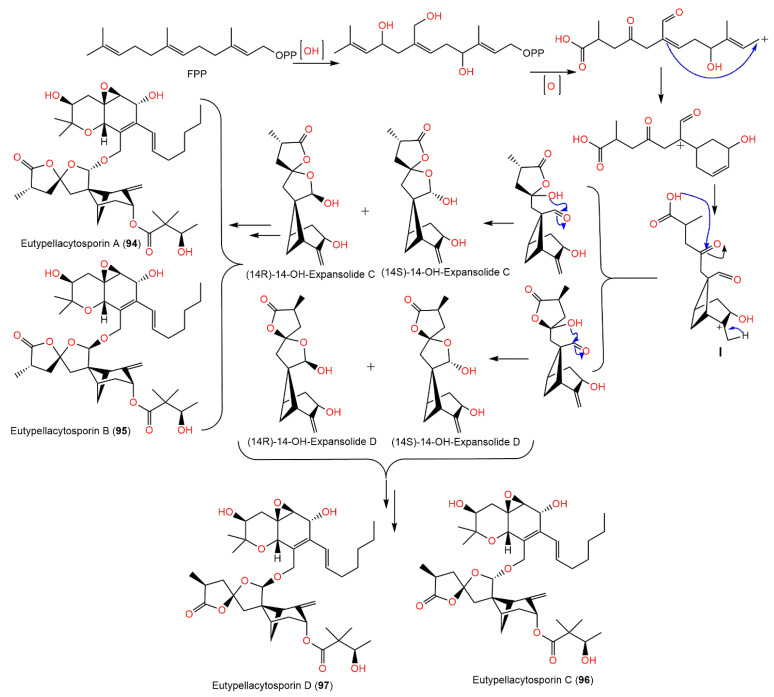
Biosynthetic pathway of eutypellacytosporins A–D (**94**–**97**) [52].

The chemical investigation of Arctic fungus *Eutypella* sp. D-1′s EtOAc extract yielded new derivatives, eutypellacytosporins A–D (**94**–**97**), which were established by spectroscopic analysis and modified Mosher’s method. Structurally, these metabolites are related to decipienolides and cytosporins. They exhibited (IC_50_s ranging from 4.9 to 17.1 μM) weak-to-moderate cytotoxic influence versus DU145, SW1990, Huh7, and PANC-1 in the CCK-8 assay, whereas Huh7 and SW1990 cell lines had more sensitivity to **94**–**97** (IC_50_s ranging from 4.9 to 8.4 μM). On the other hand, compounds **95** and **97** possessed noticeable potential versus PANC-1 (IC_50_s of 7.9 and 7.5 μM, respectively) compared with cisplatin (IC_50_ 4.5 μM). The results revealed that the decipienolide moiety was substantial for activity; however, the C-33 configuration did not affect the activity [52]. It was proposed that compounds **94**–**97** are created from gentisaldehyde precursor with subsequent isoprenyl unit addition, double bond epoxidation, keto group hydrogenation, and an aliphatic chain addition (Scheme 6). The other precursor, the 14-OH of decipienolide A **74** or B **75**, is produced from hydroxylation, allylic oxidation, and cyclization of farnesyl diphosphate to give **I** with a bicycle[3.1.1]heptane. Additionally, (14S)-14-OH-expansolide C, (14R)-14-OH-expansolide C, (14S)-14-OH-expansolide D, and (14R)-14-OH-expansolide D are formed via two steps of reface- and si-face attacks of the OH groups on the ketone and aldehyde groups, respectively. After these steps, compounds **94**–**97** were produced from the two groups of 14-OH-expansolides C and D through condensation reactions with (S)-3-hydroxy-2,2-dimethylbutanoic acid and cytosporin D, respectively [52].

## 4. Conclusions

**Figure 11:**
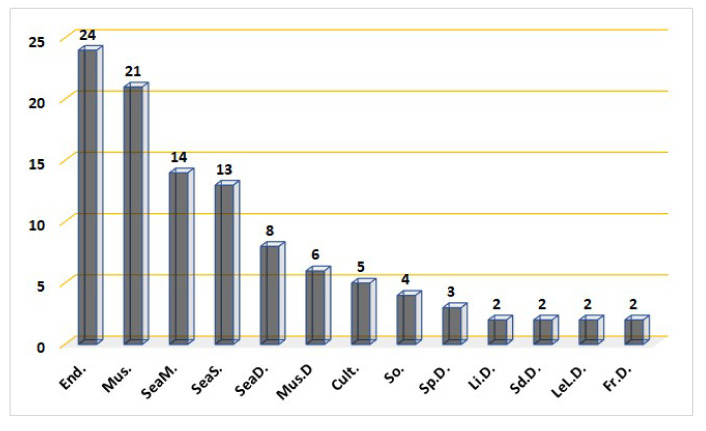
Number of bergamotane sesquiterpenoids reported from fungi derived from various sources. End.: endophytic; Mus.: mushroom; SeaM.: sea mud-derived; SeaS.: sea sediment-derived; SeaD.: sea deposit-derived; Mus.D: mushroom-derived; Cult.: cultured; So.: soil-derived; Sp.D.: sponge-derived; Li.D.: lichen-derived; Sd.D.: sheep dung-derived; LeL.D.: leaf litter-derived; Fr.D.: fruit-derived.

Fungal metabolites are an unparalleled pool for pharmaceutical lead discovery. Sesquiterpenoids involving the bergamotane skeleton have been separated from various sources, including fungi. In the current work, 97 bergamotane sesquiterpenoids were reported from various fungal species derived from different sources, including endophytic (24 compounds), mushroom (21 compounds), sea mud (14 compounds), sea sediment (13 compounds), deep-sea deposit (8 compounds), and sponges (3 compounds) (Figure 11).

**Figure 12:**
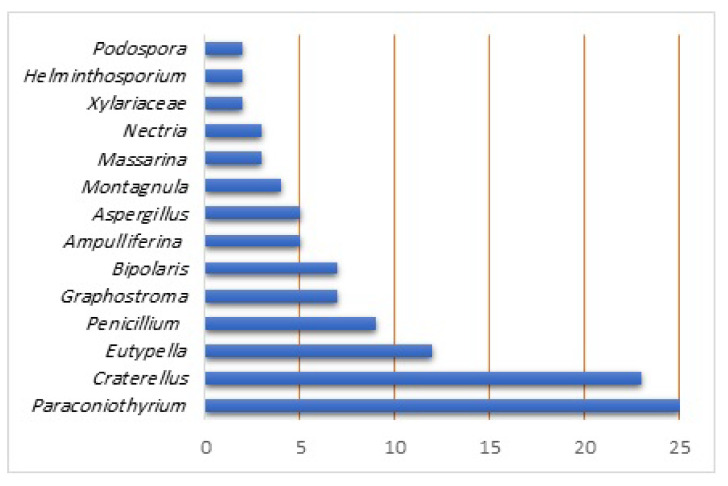
Bergamotane sesquiterpenoids from various fungal species.

The majority of compounds have been reported from *Paraconiothyrium* (25 compounds), *Craterellus* (23 compounds), and *Eutypella* (12 compounds) species (Figure 12). Interestingly, many of these metabolites normally occurred as inseparable mixtures. These metabolites were assessed for diverse bio-activities. It is obvious that cytotoxic evaluation accounts for the largest proportion of biological assessments, where they had weak or no effectiveness on the tested cell lines. On the other hand, there are limited reports on their phytotoxic, plant growth regulation, antimicrobial, anti-HIV, cytotoxic, anti-inflammatory, pancreatic lipase inhibition, immunosuppressive, and antidiabetic activities. Therefore, this suggested more potential for trying other types of pharmacological effectiveness. Victoxinine (**40**) and prehelminthosporolactone (**39**) displayed potential phytotoxic capacities; therefore, they could be utilized as bioherbicides or as lead metabolites for synthesizing more efficacious phytotoxic compounds against various weeds. Pinthunamide (**44**), ampullicin (**91**), isoampullicin (**92**), and dihydroampullicin (**93**) were found to selectively promote the root growth. However, the phytotoxic and plant growth promotion potential should be transferred from laboratory experiments into field settings for assessing the environmental influences on these activities. Purpurolide F (**87**) had potent pancreatic lipase inhibition potential that could be a viable candidate as a pancreatic lipase inhibitor for further clinical development. Massarinolin B (**61**) had prominent immunosuppressive potential, suggesting further in vivo and mechanistic investigations for the development of this metabolite as an immunosuppressant. In silico studies for the reported metabolites that have not been tested or have had no noticeable effectiveness in the estimated activities could be a possible area of future research. Moreover, synthesis and structural modifications of these metabolites may produce more potential and useful tags of these metabolites through click chemistry, which is a new approach for synthesizing drug-like molecules that can boost the drug discovery process.

Biogenetically, these metabolites are generated from acyclic farnesyl-diphosphate, which undertakes various condensation and rearrangement reactions. This work could be a beneficial reference for researchers studying this class of fungal metabolites. Several strategies, including co-culture, molecular and epigenetic manipulations, OSMAC (one strain many compounds), heterologous gene expression, and inter-species cross-talk approaches could be successively employed to access undescribed natural metabolites from silent biosynthetic pathways. It was found that the selective epigenetic target manipulation utilizing small molecule inhibitors toward DNA methyltransferase and histone deacetylase activities resulted in the enhancement of biosynthetic pathway expression for new secondary metabolite production. Highlighting the biosynthesis of these metabolites in this review could draw the attention of molecular biologists and genetics-interested researchers for isolating genes accountable for the biosynthesis of these interesting metabolites; this could allow for the discovery of the detailed mechanisms of their formation by various enzymes, which could allow for the preparation these metabolites and their analogs by engineering their biosynthetic pathways.

## Figures and Tables

**Table 1:** Naturally occurring fungal bergamotane sesquiterpenoids (name, source, extract/fraction, molecular weights and formulae, and location).

Compound Name	Fungal Source/Host	**Extract/Fraction**	**Mol. Wt.**	**Mol. Formula**	**Location**	**Ref.**
Bicyclic Bergamotane Sesquiterpenoids						
α-*trans* Bergamotene (**1**)	*Nectria* sp. HLS206 (Nectriaceae)/*Gelliodas carnosa* (marine sponge Geodiidae)	EtOAc extract	204	C_15_H_24_	China	[27]
β-*trans* Bergamotene (**2**)	*Aspergillus fumigatus* (Trichocomaceae)/Cultured	Acetone extract	204	C_15_H_24_	Japan	[28]
β-*trans*-2β,5,15-Trihydroxybergamot-10-ene (**3**)	*Aspergillus fumigatus* YK-7 (Trichocomaceae)/Sea mud	EtOAc extract	254	C_15_H_26_O_3_	Intertidal zone sea mud, Yingkou, China	[29]
*E*-β-*trans*-5,8,11-Trihydroxybergamot-9-ene (**4**)	*Aspergillus fumigatus* YK-7 (Trichocomaceae)/Sea mud	EtOAc extract	252	C_15_H_24_O_3_	Intertidal zone sea mud, Yingkou, China	[29]
Massarinolin C (**5**)	*Massarina tunicata* (Lophiostomataceae)/Submerged twig	EtOAc extract	266	C_15_H_22_O_4_	Lemonweir River in Adams County, Wisconsin, USA	[23]
	*Craterellus odoratus* (Cantharellaceae)	EtOAc extract	-	-	Southern part of the GaoligongMountains, Yunnan, China	[30]
	*Paraconiothyrium sporulosum* YK-03 Verkley (Leptosphaeriaceae)/Sea mud	EtOAc extract	-	-	Intertidal zone of Bohai Bay river in Liaoning, China	[31]
Donacinoic acid B (**6**)	*Montagnula donacina* (Montagnulaceae)/*Craterellus odoratus* (fruiting bodies, Cantharellaceae)	EtOAc extract	266	C_15_H_22_O_4_	Southern part of the Gaoligong Mountains in Yunnan, China	[32]
Craterodoratin M (**7**)	*Craterellus odoratus* (Cantharellaceae)	EtOAc extract	252	C_15_H_24_O_3_	Southern part of the GaoligongMountains, Yunnan, China	[30]
Craterodoratin N (**8**)	*Craterellus odoratus* (Cantharellaceae)	EtOAc extract	268	C_15_H_24_O_4_	Southern part of the GaoligongMountains, Yunnan, China	[30]
Craterodoratin O (**9**)	*Craterellus odoratus* (Cantharellaceae)	EtOAc extract	250	C_15_H_22_O_3_	Southern part of the GaoligongMountains, Yunnan, China	[30]
Craterodoratin P (**10**)	*Craterellus odoratus* (Cantharellaceae)	EtOAc extract	250	C_15_H_22_O_3_	Southern part of the GaoligongMountains, Yunnan, China	[30]
Craterodoratin Q (**11**)	*Craterellus odoratus* (Cantharellaceae)	EtOAc extract	308	C_17_H_24_O_5_	Southern part of the GaoligongMountains, Yunnan, China	[30]
Necbergamotenoic acid A (**12**)	*Nectria* sp. HLS206 (Nectriaceae)/*Gelliodas carnosa* (marine sponge, Geodiidae)	EtOAc extract	264	C_15_H_20_O_4_	China	[27]
Necbergamotenoic acid B (**13**)	*Nectria* sp. HLS206 (Nectriaceae)/*Gelliodas carnosa* (marine sponge, Geodiidae)	EtOAc extract	266	C_15_H_22_O_4_	China	[27]
Sporulamide C (**14**)	*Paraconiothyrium sporulosum* YK-03 Verkley (Leptosphaeriaceae)/Sea mud	EtOAc extract	265	C_15_H_23_NO_3_	Intertidal zone of Bohai Bay river in Liaoning, China	[31]
Sporulamide D (**15**)	*Paraconiothyrium sporulosum* YK-03 Verkley (Leptosphaeriaceae)/Sea mud	EtOAc extract	249	C_15_H_23_NO_2_	Intertidal zone of Bohai Bay river in Liaoning, China	[31]
Xylariterpenoid A (**16**)	*Xylariaceae fungus* (No. 63-19-7-3)/*Everniastrum cirrhatum* (Fr.) Haleex Sipman (lichen, Parmeliaceae)	EtOAc extract	252	C_15_H_24_O_3_	Zixi Mountain, Yunnan, China	[33]
	*Graphostroma* sp. MCCC 3A00421/Deep-sea hydrothermal sulfide deposit	EtOAc extract	-	-	Atlantic Ocean, China	[34]
	*Eutypella* sp. MCCC 3A00281 (Diatrypaceae)/Deep-sea sediment	EtOAc extract	-	-	South Atlantic Ocean, China	[35]
Xylariterpenoid B (**17**)	*Xylariaceae fungus* (No. 63-19-7-3)/*Everniastrum cirrhatum* (Fr.) Haleex Sipman (lichen, Parmeliaceae)	EtOAc extract	252	C_15_H_24_O_3_	Zixi Mountain, Yunnan, China	[33]
	*Graphostroma* sp. MCCC 3A00421/Deep-sea hydrothermal sulfide deposit	EtOAc extract	-	-	Atlantic Ocean, China	[34]
	*Eutypella* sp. MCCC 3A00281 (Diatrypaceae)/Deep-sea sediment	EtOAc extract	-	-	South Atlantic Ocean, China	[35]
Eutypeterpene B (**18**)	*Eutypella* sp. MCCC 3A00281 (Diatrypaceae)/Deep-sea sediment	EtOAc extract	268	C_15_H_24_O_4_	South Atlantic Ocean, China	[35]
Eutypeterpene C (**19**)	*Eutypella* sp. MCCC 3A00281 (Diatrypaceae)/Deep-sea sediment	EtOAc extract	266	C_15_H_22_O_4_	South Atlantic Ocean, China	[35]
Eutypeterpene D (**20**)	*Eutypella* sp. MCCC 3A00281 (Diatrypaceae)/Deep-sea sediment	EtOAc extract	250	C_15_H_22_O_3_	South Atlantic Ocean, China	[35]
Eutypeterpene E (**21**)	*Eutypella* sp. MCCC 3A00281 (Diatrypaceae)/Deep-sea sediment	EtOAc extract	250	C_15_H_22_O_3_	South Atlantic Ocean, China	[35]
Eutypeterpene F (**22**)	*Eutypella* sp. MCCC 3A00281 (Diatrypaceae)/Deep-sea sediment	EtOAc extract	252	C_15_H_24_O_3_	South Atlantic Ocean, China	[35]
(10S)-Xylariterpenoid A (**23**)	*Graphostroma* sp. MCCC 3A00421/Deep-sea hydrothermal sulfide deposit	EtOAc extract	252	C_15_H_24_O_3_	Atlantic Ocean. China	[34]
(10R)-Xylariterpenoid B (**24**)	*Graphostroma* sp. MCCC 3A00421/Deep-sea hydrothermal sulfide deposit	EtOAc extract	252	C_15_H_24_O_3_	Atlantic Ocean. China	[34]
Xylariterpenoid E (**25**)	*Graphostroma* sp. MCCC 3A00421/Deep-sea hydrothermal sulfide deposit	EtOAc extract	208	C_12_H_16_O_3_	Atlantic Ocean. China	[34]
Xylariterpenoid F (**26**)	*Graphostroma* sp. MCCC 3A00421/Deep-sea hydrothermal sulfide deposit	EtOAc extract	270	C_15_H_26_O_4_	Atlantic Ocean. China	[34]
Xylariterpenoid G (**27**)	*Graphostroma* sp. MCCC 3A00421/Deep-sea hydrothermal sulfide deposit	EtOAc extract	270	C_15_H_26_O_4_	Atlantic Ocean. China	[34]
Eutypeterpene A (**28**)	*Eutypella* sp. MCCC 3A00281 (Diatrypaceae)/Deep-sea sediment	EtOAc extract	294	C_16_H_22_O_5_	South Atlantic Ocean, China	[35]
Craterodoratin A (**29**)	*Craterellus odoratus* (Cantharellaceae)	EtOAc extract	252	C_15_H_24_O_3_	Southern part of the GaoligongMountains, Yunnan, China	[30]
Craterodoratin C (**30**)	*Craterellus odoratus* (Cantharellaceae)	EtOAc extract	268	C_15_H_24_O_4_	Southern part of the GaoligongMountains, Yunnan, China	[30]
Craterodoratin D (**31**)	*Craterellus odoratus* (Cantharellaceae)	EtOAc extract	268	C_15_H_24_O_4_	Southern part of the GaoligongMountains, Yunnan, China	[30]
Craterodoratin E (**32**)	*Craterellus odoratus* (Cantharellaceae)	EtOAc extract	284	C_15_H_24_O_5_	Southern part of the GaoligongMountains, Yunnan, China	[30]
Craterodoratin F (**33**)	*Craterellus odoratus* (Cantharellaceae)	EtOAc extract	284	C_15_H_24_O_5_	Southern part of the GaoligongMountains, Yunnan, China	[30]
Dihydroprehelminthosporol (**34**)	*Bipolaris* sp. No. 36/Johnson grass leaf	EtOAc extract	238	C_15_H_26_O_2_	Wake County, North Carolina, USA	[36,37]
Helminthosporal acid (**35**)	*Bipolaris* sp. No. 36/Johnson grass leaf	EtOAc extract	250	C_15_H_22_O_3_	Wake County, North Carolina, USA	[36]
Helminthosporol (**36**)	*Bipolaris* sp. No. 36/Johnson grass leaf	EtOAc extract	236	C_15_H_24_O_2_	Wake County, North Carolina, USA	[36]
Helminthosporic acid (**37**)	*Bipolaris* sp. No. 36/Johnson grass leaf	EtOAc extract	252	C_15_H_24_O_3_	Wake County, North Carolina, USA	[36]
Tricyclic Bergamotane Sesquiterpenoids						
Prehelminthosporol (**38**)	*Bipolaris* sp. No. 36/Johnson grass leaf	EtOAc extract	236	C_15_H_24_O_2_	Wake County, North Carolina, USA	[36,37]
Prehelminthosporolactone (**39**)	*Bipolaris* sp. No. 36/Johnson grass leaf	EtOAc extract	234	C_15_H_22_O_2_	Wake County, North Carolina, USA	[37]
Victoxinine (**40**)	*Helminthosporium victoriae* (Totiviridae)	Diethyl ether extract	263	C_17_H_29_NO	USA	[36,38,39]
	*Helminthosporium sativum* (Totiviridae)	Diethyl ether fraction/CHCl_3_ extract	-	-	Canada	[40]
Victoxinine-α-glycerophosphate (**41**)	*H. sativum* (Totiviridae)	*n*-BuOH extract	417	C_20_H_36_NO_6_P	USA	[41]
Craterodoratin S (**42**)	*Craterellus odoratus* (Cantharellaceae)	EtOAc extract	277	C_17_H_27_NO_2_	Southern part of the GaoligongMountains, Yunnan, China	[30]
Isosativenediol (**43**)	*Bipolaris* sp. No. 36/Johnson grass leaf	EtOAc extract	236	C_15_H_24_O_2_	Wake County, North Carolina, USA	[36]
Pinthunamide (**44**)	*Ampulliferina* sp. No. 27 (Ampullicephala)/*Pinus thunbergii* (dead tree, Pinaceae)	Acetone extract	277	C_15_H_19_NO_4_	Japan	[42]
	*Paraconiothyrium brasiliense* Verkley (M3–3341) (Leptosphaeriaceae)/ *Acer truncatum* Bunge (branches, Sapindaceae)	Acetone extract	-	-	Dongling Mountain, Beijing, China	[43]
Brasilamide A (**45**)	*Paraconiothyrium brasiliense* Verkley (M3–3341) (Leptosphaeriaceae)/ *Acer truncatum* Bunge (branches, Sapindaceae)	Acetone extract	293	C_15_H_19_NO_5_	Dongling Mountain, Beijing, China	[43,44]
Brasilamide B (**46**)	*Paraconiothyrium brasiliense* Verkley (M3–3341) (Leptosphaeriaceae)/ *Acer truncatum* Bunge (branches, Sapindaceae)	Acetone extract	265	C_15_H_23_NO_3_	Dongling Mountain, Beijing, China	[43]
Brasilamide C (**47**)	*Paraconiothyrium brasiliense* Verkley (M3–3341) (Leptosphaeriaceae)/ *Acer truncatum* Bunge (branches, Sapindaceae)	Acetone extract	279	C_15_H_21_NO_4_	Dongling Mountain, Beijing, China	[43,44]
Brasilamide D (**48**)	*Paraconiothyrium brasiliense* Verkley (M3–3341) (Leptosphaeriaceae)/ *Acer truncatum* Bunge (branches, Sapindaceae)	Acetone extract	321	C_17_H_23_NO_5_	Dongling Mountain, Beijing, China	[43]
Brasilamide K (**49**)	*Paraconiothyrium brasiliense* Verkley (M3–3341) (Leptosphaeriaceae)/ *Acer truncatum* Bunge (branches, Sapindaceae)	EtOAc extract	279	C_15_H_21_NO_4_	Dongling Mountain, Beijing, China	[44]
Brasilamide L (**50**)	*Paraconiothyrium brasiliense* Verkley (M3–3341) (Leptosphaeriaceae)/ *Acer truncatum* Bunge (branches, Sapindaceae)	EtOAc extract	265	C_15_H_23_NO_3_	Dongling Mountain, Beijing, China	[44]
Brasilamide M (**51**)	*Paraconiothyrium brasiliense* Verkley (M3–3341) (Leptosphaeriaceae)/ *Acer truncatum* Bunge (branches, Sapindaceae)	EtOAc extract	293	C_15_H_19_NO_5_	Dongling Mountain, Beijing, China,	[44]
Brasilamide N (**52**)	*Paraconiothyrium brasiliense* Verkley (M3–3341) (Leptosphaeriaceae)/ *Acer truncatum* Bunge (branches, Sapindaceae)	EtOAc extract	279	C_15_H_21_NO_4_	Dongling Mountain, Beijing, China	[44]
Craterodoratin I (**53**)	*Craterellus odoratus* (Cantharellaceae)	EtOAc extract	250	C_15_H_22_O_3_	Southern part of the GaoligongMountains, Yunnan, China	[30]
Craterodoratin J (**54**)	*Craterellus odoratus* (Cantharellaceae)	EtOAc extract	282	C_15_H_22_O_5_	Southern part of the GaoligongMountains, Yunnan, China	[30]
Craterodoratin K (**55**)	*Craterellus odoratus* (Cantharellaceae)	EtOAc extract	282	C_15_H_22_O_5_	Southern part of the GaoligongMountains, Yunnan, China	[30]
Craterodoratin L (**56**)	*Craterellus odoratus* (Cantharellaceae)	EtOAc extract	278	C_15_H_18_O_5_	Southern part of the GaoligongMountains, Yunnan, China	[30]
Sporulosoic acid A (**57**)	*Paraconiothyrium sporulosum* YK-03 Verkley (Leptosphaeriaceae)/Sea mud	EtOAc extract	282	C_15_H_22_O_5_	Intertidal zone of Bohai Bay river in Liaoning, China	[31]
Sporulosoic acid B (**58**)	*Paraconiothyrium sporulosum* YK-03 Verkley (Leptosphaeriaceae)/Sea mud	EtOAc extract	280	C_15_H_20_O_5_	Intertidal zone of Bohai Bay river in Liaoning, China	[31]
Sporulamide A (**59**)	*Paraconiothyrium sporulosum* YK-03 Verkley (Leptosphaeriaceae)/Sea mud	EtOAc extract	265	C_15_H_23_NO_3_	Intertidal zone of Bohai Bay river in Liaoning, China	[31]
Sporulamide B (**60**)	*Paraconiothyrium sporulosum* YK-03 Verkley (Leptosphaeriaceae)/Sea mud	EtOAc extract	249	C_15_H_23_NO_2_	Intertidal zone of Bohai Bay river in Liaoning, China	[31]
Massarinolin B (**61**)	*Massarina tunicata* (Lophiostomataceae)/Submerged twig	EtOAc extract	266	C_15_H_22_O_4_	Lemonweir River in Adams County, Wisconsin, USA	[23]
	*Craterellus odoratus* (Cantharellaceae)	EtOAc extract	-	-	Southern part of the GaoligongMountains, Yunnan, China	[30]
Massarinolin B methyl ester (**62**)	*Paraconiothyrium sporulosum* YK-03 Verkley (Leptosphaeriaceae)/Sea mud	EtOAc extract	280	C_16_H_24_O_4_	Intertidal zone of Bohai Bay river in Liaoning, China	[31]
Craterodoratin R (**63**)	*Craterellus odoratus* (Cantharellaceae)	EtOAc extract	282	C_15_H_22_O_5_	Southern part of the GaoligongMountains, Yunnan, China	[30]
Craterodoratin G (**64**)	*Craterellus odoratus* (Cantharellaceae)	EtOAc extract	278	C_16_H_22_O_4_	Southern part of the GaoligongMountains, Yunnan, China	[30]
Craterodoratin H (**65**)	*Craterellus odoratus* (Cantharellaceae)	EtOAc extract	278	C_16_H_22_O_4_	Southern part of the GaoligongMountains, Yunnan, China	[30]
Brasilterpene A (**66**)	*Paraconiothyrium brasiliense* HDN15-135 (Leptosphaeriaceae)/Deep-sea sediment	EtOAc extract	294	C_16_H_22_O_5_	Indian Ocean, China	[45]
Brasilterpene B (**67**)	*Paraconiothyrium brasiliense* HDN15-135 (Leptosphaeriaceae)/Deep-sea sediment	EtOAc extract	294	C_16_H_22_O_5_	Indian Ocean, China	[45]
Brasilterpene C (**68**)	*Paraconiothyrium brasiliense* HDN15-135 (Leptosphaeriaceae)/Deep-sea sediment	EtOAc extract	278	C_16_H_22_O_4_	Indian Ocean, China	[45]
Brasilterpene D (**69**)	*Paraconiothyrium brasiliense* HDN15-135 (Leptosphaeriaceae)/Deep-sea sediment	EtOAc extract	278	C_16_H_22_O_4_	Indian Ocean, China	[45]
Brasilterpene E (**70**)	*Paraconiothyrium brasiliense* HDN15-135 (Leptosphaeriaceae)/Deep-sea sediment	EtOAc extract	278	C_16_H_22_O_4_	Indian Ocean, China	[45]
Craterodoratin B (**71**)	*Craterellus odoratus* (Cantharellaceae)	EtOAc extract	266	C_15_H_22_O_4_	Southern part of the GaoligongMountains, Yunnan, China	[30]
Tetracyclic Bergamotane Sesquiterpenoids						
Expansolide A (**72**)	*Penicillium expansum* (Trichocomaceae)/Fruit	EtOAc extract	306	C_17_H_22_O_5_	France	[25]
	*Aspergillus fumigatus* Fresenius (Trichocomaceae)/Leaf litter	EtOAc extract	-	-	Waipoua Forest, New Zealand	[26]
Expansolide C (**73**)	*Penicillium expansum* ACCC37275/Agricultural Culture	Acetone extract	264	C_15_H_20_O_4_	China	[46]
Decipienolide A (**74**)	*Podospora decipiens* Niessl (JS 270) (Podosporaceae)/Sheep dung	EtOAc extract	378	C_21_H_30_O_6_	South Australia	[24]
Decipienolide B (**75**)	*Podospora decipiens* Niessl (JS 270) (Podosporaceae)/Sheep dung	EtOAc extract	378	C_21_H_30_O_6_	South Australia	[24]
Donacinolide B (**76**)	*Montagnula donacina* (Montagnulaceae)/*Craterellus odoratus* (fruiting bodies, Cantharellaceae)	EtOAc extract	246	C_15_H_18_O_3_	Southern part of the Gaoligong Mountains in Yunnan, China	[32]
Massarinolin A (**77**)	*Massarina tunicata* (Lophiostomataceae)/Submerged twig	EtOAc extract	262	C_15_H_18_O_4_	LemonweirRiver in Adams County, Wisconsin, USA	[23]
	*Craterellus odoratus* (Cantharellaceae)	EtOAc extract	-	-	Southern part of the GaoligongMountains, Yunnan, China	[30]
Sporuloketal A (**78**)	*Paraconiothyrium sporulosum* YK-03 Verkley (Leptosphaeriaceae)/Sea mud	EtOAc extract	262	C_15_H_18_O_4_	Intertidal zone of Bohai Bay river in Liaoning, China	[31]
Sporuloketal B (**79**)	*Paraconiothyrium sporulosum* YK-03 Verkley (Leptosphaeriaceae)/Sea mud	EtOAc extract	262	C_15_H_18_O_4_	Intertidal zone of Bohai Bay river in Liaoning, China	[31]
Expansolide B (**80**)	*Penicillium expansum* (Trichocomaceae)	EtOAc extract	306	C_17_H_22_O_5_	France	[25]
	*Aspergillus fumigatus* Fresenius (Trichocomaceae)/Leaf litter	EtOAc extract	-	-	Waipoua Forest, New Zealand	[26]
Expansolide D (**81**)	*Penicillium expansum* ACCC37275, (Trichocomaceae)/Agricultural Culture	Acetone extract	264	C_15_H_20_O_4_	China	[46]
Donacinolide A (**82**)	*Montagnula donacina* (Montagnulaceae)/*Craterellus odoratus* (fruiting bodies, Cantharellaceae)	EtOAc extract	246	C_15_H_18_O_3_	Southern part of the Gaoligong Mountains in Yunnan, China	[32]
Purpurolide B (**83**)	*Penicillium purpurogenum* IMM003 (Trichocomaceae)/*Edgeworthia Chrysantha* (leaves, Thymelaeaceae)	EtOAc extract	336	C_17_H_20_O_7_	Hangzhou Bay, Hangzhou, Zhejiang, China	[47]
Purpurolide C (**84**)	*Penicillium purpurogenum* IMM003 (Trichocomaceae)/*Edgeworthia Chrysantha* (leaves, Thymelaeaceae)	EtOAc extract	308	C_16_H_20_O_6_	Hangzhou Bay, Hangzhou, Zhejiang, China	[47]
Purpurolide D (**85**)	*Penicillium purpurogenum* IMM003 (Trichocomaceae)/*Edgeworthia Chrysantha* (leaves, Thymelaeaceae)	EtOAc extract	294	C_15_H_18_O_6_	Hangzhou Bay, Hangzhou, Zhejiang, China	[48]
Purpurolide E (**86**)	*Penicillium purpurogenum* IMM003 (Trichocomaceae)/*Edgeworthia Chrysantha* (leaves, Thymelaeaceae)	EtOAc extract	278	C_15_H_18_O_5_	Hangzhou Bay, Hangzhou, Zhejiang, China	[48]
Purpurolide F (**87**)	*Penicillium purpurogenum* IMM003 (Trichocomaceae)/*Edgeworthia Chrysantha* (leaves, Thymelaeaceae)	EtOAc extract	464	C_25_H_36_O_8_	Hangzhou Bay, Hangzhou, Zhejiang, China	[48]
Donacinoic acid A (**88**)	*Montagnula donacina* (Montagnulaceae)/*Craterellus odoratus* (fruiting bodies, Cantharellaceae)	EtOAc extract	264	C_15_H_20_O_4_	Southern part of the Gaoligong Mountains in Yunnan, China	[32]
	*Craterellus odoratus* (Cantharellaceae)	EtOAc extract	-	-	Southern part of the GaoligongMountains, Yunnan, China	[30]
Sporulaminal A (**89**)	*Paraconiothyrium sporulosum* YK-03 (Leptosphaeriaceae)/Sea mud	EtOAc extract	247	C_15_H_21_NO_2_	Intertidal zone of Bohai river in Liaonign, China	[49]
Sporulaminal B (**90**)	*Paraconiothyrium sporulosum* YK-03 (Leptosphaeriaceae)/Sea mud	EtOAc extract	247	C_15_H_21_NO_2_	Intertidal zone of Bohai river in Liaonign, China	[49]
Ampullicin (**91**)	*Ampulliferina*-like sp. No. 27 (Ampullicephala)/*Pinus thunbergii* (dead tree, Pinaceae)	Acetone extract	259	C_15_H_17_NO_3_	Japan	[50,51]
Isoampullicin (**92**)	*Ampulliferina*-like sp. No. 27 (Ampullicephala)/*Pinus thunbergii* (dead tree, Pinaceae)	Acetone extract	259	C_15_H_17_NO_3_	Japan	[50]
Dihydroampullicin (**93**)	*Ampulliferina*-like sp. No. 27 (Ampullicephala)/*Pinus thunbergii* (dead tree, Pinaceae)	Acetone extract	261	C_15_H_19_NO_3_	Japan	[51]
Eutypellacytosporin A (**94**)	*Eutypella* sp. D-1 (Diatrypaceae)/Soil sample	CH_2_Cl_2_ fraction of EtOAc extract	714	C_40_H_58_O_11_	London Island of Kongsfjorden of the Ny-Ålesund District, Arctic, Norway	[52]
Eutypellacytosporin B (**95**)	*Eutypella* sp. D-1 (Diatrypaceae)/Soil sample	CH_2_Cl_2_ fraction of EtOAc extract	714	C_40_H_58_O_11_	London Island of Kongsfjorden of the Ny-Ålesund District, Arctic, Norway	[52]
Eutypellacytosporin C (**96**)	*Eutypella* sp. D-1 (Diatrypaceae)/Soil sample	CH_2_Cl_2_ fraction of EtOAc extract	714	C_40_H_58_O_11_	London Island of Kongsfjorden of the Ny-Ålesund District, Arctic, Norway	[52]
Eutypellacytosporin D (**97**)	*Eutypella* sp. D-1 (Diatrypaceae)/Soil sample	CH_2_Cl_2_ fraction of EtOAc extract	714	C_40_H_58_O_11_	London Island of Kongsfjorden of the Ny-Ålesund District, Arctic, Norway	[52]

**Table 2:** Biological activities of fungal naturally occurring in bergamotane sesquiterpenoids.

Compound Name	**Biological Activity**	**Assay, Organism, or Cell Line**	**Biological Results**		**Ref.**
			**Compound**	**Positive Control**	
E-β-*trans*-5,8,11-trihydroxybergamot-9-ene (**4**)	Cytotoxicity	MTT/U937	84.9 (IC_50_)	Doxorubicin 0.021 µM (IC_50_)	[29]
Craterodoratin M (**7**)	Immunosuppressive	BALB/c mice T and B lymphocyte/LPS	15.43 μM (IC_50_)	Cyclosporin A 0.47 μM (IC_50_)	[30]
Craterodoratin N (**8**)	Immunosuppressive	BALB/c mice T and B lymphocyte/LPS	13.26 μM (IC_50_)	Cyclosporin A 0.47 μM (IC_50_)	[30]
Craterodoratin O (**9**)	Immunosuppressive	BALB/c mice T and B lymphocyte/LPS	17.12 μM (IC_50_)	Cyclosporin A 0.47 μM (IC_50_)	[30]
Craterodoratin Q (**11**)	Immunosuppressive	BALB/c mice T and B lymphocyte/Concanavalin A	31.50 μM (IC_50_)	Cyclosporin A 0.04 μM (IC_50_)	[30]
Xylariterpenoid A (**16**)	Anti-inflammatory	Spectrophotometrically/LPS	17.5 μM (IC_50_)	Quercetin 17.0 μM (IC_50_) NG-monomethyl-L-arginine 9.7 μM (IC_50_)	[35]
Xylariterpenoid B (**17**)	Anti-inflammatory	Spectrophotometrically/LPS	21.0 μM (IC_50_)	Quercetin 17.0 μM (IC_50_) NG-monomethyl-L-arginine 9.7 μM (IC_50_)	[35]
Eutypeterpene B (**18**)	Anti-inflammatory	Spectrophotometrically/LPS	13.4 μM (IC_50_)	Quercetin 17.0 μM (IC_50_) NG-monomethyl-L-arginine 9.7 μM (IC_50_)	[35]
Eutypeterpene C (**19**)	Anti-inflammatory	Spectrophotometrically/LPS	16.8 μM (IC_50_)	Quercetin 17.0 μM (IC_50_) NG-monomethyl-L-arginine 9.7 μM (IC_50_)	[35]
Eutypeterpene D (**20**)	Anti-inflammatory	Spectrophotometrically/LPS	21.4 μM (IC_50_)	Quercetin 17.0 μM (IC_50_) NG-monomethyl-L-arginine 9.7 μM (IC_50_)	[35]
Eutypeterpene E (**21**)	Anti-inflammatory	Spectrophotometrically/LPS	18.7 μM (IC_50_)	Quercetin 17.0 μM (IC_50_) NG-monomethyl-L-arginine 9.7 μM (IC_50_)	[35]
Eutypeterpene F (**22**)	Anti-inflammatory	Spectrophotometrically/LPS	24.3 μM (IC_50_)	Quercetin 17.0 μM (IC_50_) NG-monomethyl-L-arginine 9.7 μM (IC_50_)	[35]
(10S)-Xylariterpenoid A (**23**)	Anti-inflammatory	Spectrophotometrically/LPS	86.0 μM (IC_50_)	Aminoguanidine 23.0 μM (IC_50_)	[34]
(10R)-Xylariterpenoid B (**24**)	Anti-inflammatory	Spectrophotometrically/LPS	230.0 μM (IC_50_)	Aminoguanidine 23.0 μM (IC_50_)	[34]
Xylariterpenoid E (**25**)	Anti-inflammatory	Spectrophotometrically/LPS	120.0 μM (IC_50_)	Aminoguanidine 23.0 μM (IC_50_)	[34]
Xylariterpenoid F (**26**)	Anti-inflammatory	Spectrophotometrically/LPS	85.0 μM (IC_50_)	Aminoguanidine 23.0 μM (IC_50_)	[34]
Xylariterpenoid G (**27**)	Anti-inflammatory	Spectrophotometrically/LPS	85.0 μM (IC_50_)	Aminoguanidine 23.0 μM (IC_50_)	[34]
Eutypeterpene A (**28**)	Anti-inflammatory	Spectrophotometrically/LPS	21.0 μM (IC_50_)	Quercetin 17.0 μM (IC_50_) NG-monomethyl-L-arginine 9.7 μM (IC_50_)	[35]
Craterodoratin C (**30**)	Immunosuppressive	BALB/c mice T and B lymphocyte/LPS	12.62 μM (IC_50_)	Cyclosporin A 0.47 μM (IC_50_)	[30]
Craterodoratin S (**42**)	Immunosuppressive	BALB/c mice T and B lymphocyte/LPS	22.68 μM (IC_50_)	Cyclosporin A 0.47 μM (IC_50_)	[30]
Craterodoratin J (**54**)	Immunosuppressive	BALB/c mice T and B lymphocyte/LPS	19.40 μM (IC_50_)	Cyclosporin A 0.47 μM (IC_50_)	[30]
Craterodoratin L (**56**)	Immunosuppressive	BALB/c mice T and B lymphocyte/LPS	13.71 μM (IC_50_)	Cyclosporin A 0.47 μM (IC_50_)	[30]
Massarinolin B (**61**)	Immunosuppressive	BALB/c mice T and B lymphocyte/Concanavalin A	0.98 μM (IC_50_)	Cyclosporin A 0.04 μM (IC_50_)	[30]
Brasilterpene A (**66**)	Hypoglycemic	Spectrophotometrically/Diabetic zebrafish model	449.3 pmol/larva (IC_50_)	Rosiglitazone 395.6 pmol/larva (IC_50_)	[45]
Brasilterpene C (**68**)	Hypoglycemic	Spectrophotometrically/Diabetic zebrafish model	420.4 pmol/larva (IC_50_)	Rosiglitazone 395.6 pmol/larva (IC_50_)	[45]
Expansolide C (**73**)	α-Glucosidase inhibition	Spectrophotometrically/α-glucosidase enzyme	0.50 mM (IC_50_)	Acarbose 1.90 mM (IC_50_)	[46]
Expansolide D (**81**)	α-Glucosidase inhibition	Spectrophotometrically/α-glucosidase enzyme	0.50 mM (IC_50_)	acarbose 1.90 mM (IC_50_)	[46]
Purpurolide B (**83**)	Pancreatic lipase inhibition	Spectrophotometrically/pancreatic lipase enzyme	5.45 μM (IC_50_)	Kaempferol 1.50 μM (IC_50_)	[47]
Purpurolide C (**84**)	Pancreatic lipase inhibition	Spectrophotometrically/pancreatic lipase enzyme	6.63 μM (IC_50_)	Kaempferol 1.50 μM (IC_50_)	[47]
Purpurolide D (**85**)	Pancreatic lipase inhibition	Spectrophotometrically/pancreatic lipase enzyme	1.22 μM (IC_50_)	Kaempferol 1.50 μM (IC_50_)	[48]
Purpurolide E (**86**)	Pancreatic lipase inhibition	Spectrophotometrically/pancreatic lipase enzyme	6.50 μM (IC_50_)	Kaempferol 1.50 μM (IC_50_)	[48]
Purpurolide F (**87**)	Pancreatic lipase inhibition	Spectrophotometrically/pancreatic lipase enzyme	7.88 μM (IC_50_)	Kaempferol 1.50 μM (IC_50_)	[48]
Donacinoic acid A (**88**)	Immunosuppressive	BALB/c mice T and B lymphocyte/LPS	13.23 μM (IC_50_)	Cyclosporin A 0.47 μM (IC_50_)	[30]
Eutypellacytosporin A (**94**)	Cytotoxicity	CCK-8/DU145	17.1 μM (IC_50_)	Cisplatin 2.9 μM (IC_50_)	[52]
		CCK-8/SW1990	7.3 μM (IC_50_)	Cisplatin 1.2 μM (IC_50_)	[52]
		CCK-8/Huh7	8.4 μM (IC_50_)	Cisplatin 2.2 μM (IC_50_)	[52]
		CCK-8/PANC-1	9.7 μM (IC_50_)	Cisplatin 4.5 μM (IC_50_)	[52]
Eutypellacytosporin B (**95**)	Cytotoxicity	CCK-8/DU145	11.0 μM (IC_50_)	Cisplatin 2.9 μM (IC_50_)	[52]
		CCK-8/SW1990	4.9 μM (IC_50_)	Cisplatin 1.2 μM (IC_50_)	[52]
		CCK-8/Huh7	4.9 μM (IC_50_)	Cisplatin 2.2 μM (IC_50_)	[52]
		CCK-8/PANC-1	7.9 μM (IC_50_)	Cisplatin 4.5 μM (IC_50_)	[52]
Eutypellacytosporin C (**96**)	Cytotoxicity	CCK-8/DU145	13.5 μM (IC_50_)	Cisplatin 2.9 μM (IC_50_)	[52]
		CCK-8/SW1990	9.6 μM (IC_50_)	Cisplatin 1.2 μM (IC_50_)	[52]
		CCK-8/Huh7	11.2 μM (IC_50_)	Cisplatin 2.2 μM (IC_50_)	[52]
		CCK-8/PANC-1	10.2 μM (IC_50_)	Cisplatin 4.5 μM (IC_50_)	[52]
Eutypellacytosporin D (**97**)	Cytotoxicity	CCK-8/DU145	13.4 μM (IC_50_)	Cisplatin 2.9 μM (IC_50_)	[52]
		CCK-8/SW1990	8.2 μM (IC_50_)	Cisplatin 1.2 μM (IC_50_)	[52]
		CCK-8/Huh7	9.6 μM (IC_50_)	Cisplatin 2.2 μM (IC_50_)	[52]
		CCK-8/PANC-1	7.5 μM (IC_50_)	Cisplatin 4.5 μM (IC_50_)	[52]

## Data Availability

Not applicable.

## Abbreviations

5-Aza	5-azacytidine
A2780	Human ovarian cancer cell line
4-MUO	4-methylumbelliferyl oleate
A549	Lung adenocarcinoma epithelial cell line
Bel-7402	Human hepatoma cell line
BGC-823	Human stomach cancer cell line
BuOH	*n*-Butanol
C8166	Human T-cell leukaemia
CC	Column chromatography
CC_50_	The 50% cytotoxic concentration
CCK-8	Cell Counting Kit-8
CHCl_3_	Chloroform
CH_2_Cl_2_	Dichloromethane
CNE1-LMP1	Stable oncoprotein LMP1 integrated nasopharyngeal carcinoma cell line
DU145	Human prostate carcinoma cell line
EC109	Human esophageal cancer cell line
ED_50_	Half-maximal effective concentration
H_2_SO_4_	Sulfuric acid
Hep3B-2	Human hepatoma carcinoma cell line
HCT-116	Human colon cancer cell line
HIV	Human immunodeficiency virus
HPLC	High-performance liquid chromatography
Huh7	Human hepatoma adenocarcinoma cell line
IR	Infrared
HL-60	Human myeloid leukemia cell line
LPS	Lipopolysaccharide
M14	Human melanoma cell line
MCF-7	Human breast cancer cell line
MGC	Human gastric cancer cell line
MTS	(3-(4,5-Dimethylthiazol-2-yl)-5-(3-carboxymethoxyphenyl)-2-(4-sulfophenyl)-2H-tetrazoliuminner salt)
MTT	3-(4,5-Dimethylthiazol-2-yl)-2,5-diphenyltetrazolium bromide
NMR	Nuclear magnetic resonance
NO	Nitric oxide
RP-18	Reversed phase-18
SBHA	Histonedeacetylase inhibitor, suberohydroxamic acid
SiO_2_	Silica gel
SMMC-7721	Hepatocellular carcinoma cell line
SW480	Colon cancer cell line
SW1990	Human pancreatic adenocarcinoma cell line
PANC-1	Human pancreatic carcinoma cell line
PC-3	Human prostate cancer cell line
TLC	Thin-layer chromatography
U937	Human leukemic monocyte lymphoma cell line

## References

[1] Shen B. (2015). A New Golden Age of Natural Products Drug Discovery. Cell.

[2] Krabberød A.K., Deutschmann I.M., Bjorbækmo M.F., Balagué V., Giner C.R., Ferrera I., Garcés E., Massana R., Gasol J.M., Logares R. (2022). Long-Term Patterns of an Interconnected Core Marine Microbiota. Environ. Microbiome.

[3] Imhoff J.F. (2016). Natural Products from Marine fungi—Still an Underrepresented Resource. Mar. Drugs.

[4] Alves A., Sousa E., Kijjoa A., Pinto M. (2020). Marine-Derived Compounds with Potential use as Cosmeceuticals and Nutricosmetics. Molecules.

[5] Ibrahim S.R., Fadil S.A., Fadil H.A., Hareeri R.H., Alolayan S.O., Abdallah H.M., Mohamed G.A. (2022). *Dactylospongia Elegans*—A Promising Drug Source: Metabolites, Bioactivities, Biosynthesis, Synthesis, and Structural-Activity Relationship. Mar. Drugs.

[6] Ibrahim S.R., Fadil S.A., Fadil H.A., Hareeri R.H., Abdallah H.M., Mohamed G.A. (2022). Genus Smenospongia: Untapped Treasure of Biometabolites—Biosynthesis, Synthesis, and Bioactivities. Molecules.

[7] Mohamed G.A., Ibrahim S.R. (2021). Untapped Potential of Marine-Associated *Cladosporium* Species: An Overview on Secondary Metabolites, Biotechnological Relevance, and Biological Activities. Mar. Drugs.

[8] Ibrahim S.R.M., Mohamed G.A., Al Haidari R.A., El-Kholy A.A., Zayed M.F., Khayat M.T. (2018). Biologically Active Fungal Depsidones: Chemistry, Biosynthesis, Structural Characterization, and Bioactivities. Fitoterapia.

[9] Omar A.M., Mohamed G.A., Ibrahim S.R.M. (2022). Chaetomugilins and Chaetoviridins-Promising Natural Metabolites: Structures, Separation, Characterization, Biosynthesis, Bioactivities, Molecular Docking, and Molecular Dynamics. J. Fungi.

[10] Ibrahim S.R.M., Sirwi A., Eid B.G., Mohamed S.G.A., Mohamed G.A. (2021). Fungal Depsides-Naturally Inspiring Molecules: Biosynthesis, Structural Characterization, and Biological Activities. Metabolites.

[11] Ibrahim S.R.M., Fadil S.A., Fadil H.A., Eshmawi B.A., Mohamed S.G.A., Mohamed G.A. (2022). Fungal Naphthalenones; Promising Metabolites for Drug Discovery: Structures, Biosynthesis, Sources, and Pharmacological Potential. Toxins.

[12] Ameen F., AlNadhari S., Al-Homaidan A.A. (2021). Marine Microorganisms as an Untapped Source of Bioactive Compounds. Saudi J. Biol. Sci..

[13] Hasan S., Ansari M.I., Ahmad A., Mishra M. (2015). Major Bioactive Metabolites from Marine Fungi: A Review. Bioinformation.

[14] Wibowo J.T., Ahmadi P., Rahmawati S.I., Bayu A., Putra M.Y., Kijjoa A. (2021). Marine-Derived Indole Alkaloids and their Biological and Pharmacological Activities. Mar. Drugs.

[15] Giddings L., Newman D.J. (2022). Extremophilic Fungi from Marine Environments: Underexplored Sources of Antitumor, Anti-Infective and Other Biologically Active Agents. Mar. Drugs.

[16] Hafez Ghoran S., Taktaz F., Ayatollahi S.A., Kijjoa A. (2022). Anthraquinones and their Analogues from Marine-Derived Fungi: Chemistry and Biological Activities. Mar. Drugs.

[17] Kramer R., Abraham W. (2012). Volatile Sesquiterpenes from Fungi: What are they Good for?. Phytochem. Rev..

[18] Gui P., Fan J., Zhu T., Fu P., Hong K., Zhu W. (2022). Sesquiterpenoids from the Mangrove-Derived Aspergillus Ustus 094102. Mar. Drugs.

[19] Dai Q., Zhang F., Feng T. (2021). Sesquiterpenoids Specially Produced by Fungi: Structures, Biological Activities, Chemical and Biosynthesis (2015–2020). J. Fungi.

[20] Minami A., Ozaki T., Liu C., Oikawa H. (2018). Cyclopentane-Forming Di/Sesterterpene Synthases: Widely Distributed Enzymes in Bacteria, Fungi, and Plants. Nat. Prod. Rep..

[21] Fraga B.M. (2013). Natural Sesquiterpenoids. Nat. Prod. Rep..

[22] Cane D.E. (1990). Enzymic Formation of Sesquiterpenes. Chem. Rev..

[23] Oh H., Gloer J.B., Shearer C.A. (1999). Massarinolins A−C: New Bioactive Sesquiterpenoids from the Aquatic Fungus *Massarina Tunicata*. J. Nat. Prod..

[24] Che Y., Gloer J.B., Koster B., Malloch D. (2002). Decipinin A and Decipienolides A and B: New Bioactive Metabolites from the Coprophilous Fungus *Podospora Decipiens*. J. Nat. Prod..

[25] Massias M., Rebuffat S., Molho L., Chiaroni A., Riche C., Bodo B. (1990). Expansolides A and B: Tetracyclic Sesquiterpene Lactones from *Penicillium Expansum*. J. Am. Chem. Soc..

[26] Macías F.A., Varela R.M., Simonet A.M., Cutler H.G., Cutler S.J., Hill R.A. (2003). Absolute Configuration of Bioactive Expansolides A and B from *Aspergillus Fumigatus* Fresenius. Tetrahedron Lett..

[27] Wen Y., Chen T., Jiang L., Li L., Guo M., Peng Y., Chen J., Pei F., Yang J., Wang R. (2022). Unusual (2R, 6R)-Bicyclo [3.1. 1] Heptane Ring Construction in Fungal A-*Trans*-Bergamotene Biosynthesis. Iscience.

[28] Nozoe S., Kobayashi H., Morisaki N. (1976). Isolation of Beta Trans Bergamotene from *Aspergillus Fumigatus*, a Fumagillin Producing Fungi. Tetrahedron Lett..

[29] Wang Y., Li D., Li Z., Sun Y., Hua H., Liu T., Bai J. (2016). Terpenoids from the Marine-Derived Fungus *Aspergillus Fumigatus* YK-7. Molecules.

[30] Dai Q., Zhang F., Li Z., He J., Feng T. (2021). Immunosuppressive Sesquiterpenoids from the Edible Mushroom *Craterellus Odoratus*. J. Fungi.

[31] Pei Y., Zhang L., Wu X., Wu H., Wang H., Wang Y., Chen G. (2022). Polyhydroxylated Bergamotane-Type Sesquiterpenoids from Cultures of *Paraconiothyrium Sporulosum* YK-03 and their Absolute Configurations. Phytochemistry.

[32] Zhao Z., Zhao K., Chen H., Bai X., Zhang L., Liu J. (2018). Terpenoids from the Mushroom-Associated Fungus *Montagnula Donacina*. Phytochemistry.

[33] Wu Z., Wu Y., Chen G., Hu D., Li X., Sun X., Guo L., Li Y., Yao X., Gao H. (2014). Xylariterpenoids A–D, Four New Sesquiterpenoids from the Xylariaceae Fungus. RSC Adv..

[34] Niu S., Xie C., Zhong T., Xu W., Luo Z., Shao Z., Yang X. (2017). Sesquiterpenes from a Deep-Sea-Derived Fungus *Graphostroma* Sp. MCCC 3A00421. Tetrahedron.

[35] Niu S., Liu D., Shao Z., Liu J., Fan A., Lin W. (2021). Chemical Epigenetic Manipulation Triggers the Production of Sesquiterpenes from the Deep-Sea Derived *Eutypella* Fungus. Phytochemistry.

[36] Pena-Rodriguez L.M., Armingeon N.A., Chilton W.S. (1988). Toxins from Weed Pathogens, I. Phytotoxins from a *Bipolaris* Pathogen of Johnson Grass. J. Nat. Prod..

[37] Pena-Rodriguez L.M., Chilton W.S. (1989). Victoxinine and Prehelminthosporolactone, Two Minor Phytotoxic Metabolites Produced by *Bipolaris* Sp., a Pathogen of Johnson Grass. J. Nat. Prod..

[38] Pringle R.B., Braun A.C. (1958). Constitution of the Toxin of *Helminthosporium Victoriae*. Nature.

[39] Dorn F., Arigoni D. (1972). Structure of Victoxinine. J. Chem. Soc. Chem. Commun..

[40] Pringle R. (1976). Comparative Biochemistry of the Phytopathogenic Fungus Helminthosporium. XVI. the Production of Victoxinine by *H. Sativum* and *H. Victoriae*. Can. J. Biochem..

[41] Kono Y., Takeuchi S., Daly J.M. (1983). Isolation and Structure of a New Victoxinine Derivative Produced by *Helminthosporium Victoriae*. Agric. Biol. Chem..

[42] Kimura Y., Nakajima H., Hamasaki T., Sugawara F., Parkanyi L., Clardy J. (1989). Pinthunamide, a New Tricyclic Sesquiterpene Amide Produced by a Fungus, *Ampullifernia* Sp.. Tetrahedron Lett..

[43] Liu L., Gao H., Chen X., Cai X., Yang L., Guo L., Yao X., Che Y. (2010). Brasilamides A–D: Sesquiterpenoids from the Plant Endophytic Fungus *Paraconiothyrium Brasiliense*. Eur. J. Org. Chem..

[44] Guo Z., Ren F., Che Y., Liu G., Liu L. (2015). New Bergamotane Sesquiterpenoids from the Plant Endophytic Fungus *Paraconiothyrium Brasiliense*. Molecules.

[45] Wang W., Shi Y., Liu Y., Zhang Y., Wu J., Zhang G., Che Q., Zhu T., Li M., Li D. (2022). Brasilterpenes A-E, Bergamotane Sesquiterpenoid Derivatives with Hypoglycemic Activity from the Deep Sea-Derived Fungus *Paraconiothyrium Brasiliense* HDN15-135. Mar Drugs.

[46] Ying Y., Fang C., Yao F., Yu Y., Shen Y., Hou Z., Wang Z., Zhang W., Shan W., Zhan Z. (2017). Bergamotane Sesquiterpenes with Alpha-glucosidase Inhibitory Activity from the Plant Pathogenic Fungus *Penicillium Expansum*. Chem. Biodivers..

[47] Wang Y., Xia G., Wang L., Ge G., Zhang H., Zhang J., Wu Y., Lin S. (2018). Purpurolide A, 5/5/5 Spirocyclic Sesquiterpene Lactone in Nature from the Endophytic Fungus *Penicillium Purpurogenum*. Org. Lett..

[48] Xia G., Wang L., Zhang J., Wu Y., Ge G., Wang Y., Lin P., Lin S. (2020). Three New Polyoxygenated Bergamotanes from the Endophytic Fungus *Penicillium Purpurogenum* IMM 003 and their Inhibitory Activity Against Pancreatic Lipase. Chin. J. Nat. Med..

[49] Zhang L., Feng B., Chen G., Li S., Sun Y., Wu H., Bai J., Hua H., Wang H., Pei Y. (2016). Sporulaminals A and B: A Pair of Unusual Epimeric Spiroaminal Derivatives from a Marine-Derived Fungus *Paraconiothyrium Sporulosum* YK-03. RSC Adv..

[50] Kimura Y., Nakajima H., Hamasaki T., Matsumoto T., Matsuda Y., Tsuneda A. (1990). Ampullicin and Isoampullicin, New Metabolites from an *Ampulliferina*-Like Fungus Sp. no. 27. Agric. Biol. Chem..

[51] Kimura Y., Matsumoto T., Nakajima H., Hamasaki T., Matsuda Y. (1993). Dihydroampullicin, a New Plant Growth Regulators Produced by the *Ampulliferina*-Like Fungus Sp. no. 27. Biosci. Biotechnol. Biochem..

[52] Zhang Y., Yu H., Xu W., Hu B., Guild A., Zhang J., Lu X., Liu X., Jiao B. (2019). Eutypellacytosporins A–D, Meroterpenoids from the Arctic Fungus *Eutypella* Sp. D-1. J. Nat. Prod..

[53] Bermejo F.A., Mateos A.F., Escribano A.M., Lago R.M., Burón L.M., López M.R., González R.R. (2006). Ti (III)-Promoted Cyclizations. Application to the Synthesis of (E)-Endo-Bergamoten-12-Oic Acids. Moth Oviposition Stimulants Isolated from Lycopersicon Hirsutum. Tetrahedron.

[54] López M.R., Bermejo F.A. (2006). Total Synthesis of ( )-Massarinolin B and ( )-4-Epi-Massarinolin B, Fungal Metabolites from Massarina Tunicata. Tetrahedron.

[55] Zhao J., Feng J., Tan Z., Liu J., Zhang M., Chen R., Xie K., Chen D., Li Y., Chen X. (2018). Bistachybotrysins A-C, Three Phenylspirodrimane Dimers with Cytotoxicity from *Stachybotrys Chartarum*. Bioorg. Med. Chem. Lett..

[56] Zamora R., Vodovotz Y., Billiar T.R. (2000). Inducible Nitric Oxide Synthase and Inflammatory Diseases. Mol. Med..

[57] Kovarik J.M., Burtin P. (2003). Immunosuppressants in Advanced Clinical Development for Organ Transplantation and Selected Autoimmune Diseases. Expert Opin. Emerg. Drugs.

[58] Klinker M.W., Lundy S.K. (2012). Multiple Mechanisms of Immune Suppression by B Lymphocytes. Mol. Med..

[59] Bermejo F.A., Rico-Ferreira R., Bamidele-Sanni S., García-Granda S. (2001). Total Synthesis of (+)-Ampullicin and (+)-Isoampullicin: Two Fungal Metabolites with Growth Regulatory Activity Isolated from Ampulliferina Sp. 27. J. Org. Chem..

